# Interspecies quorum sensing signals modulate multicellular organization and enhance contact-dependent antagonism in *Vibrio cholerae*

**DOI:** 10.1038/s41467-026-74086-w

**Published:** 2026-06-08

**Authors:** Mollie Virgo, Hannah Painter, Songlin Xue, Harry-Luke O. McClelland, Serge Mostowy, Brian T. Ho

**Affiliations:** 1 Institute of Structural and Molecular Biology, Department of Biological Sciences, Birkbeck College, London, UK; 2 Department of Infection Biology, London School of Hygiene and Tropical Medicine, London, UK; 3 Institute of Structural and Molecular Biology, Division of Biosciences, University College London, London, UK

**Keywords:** Pathogens, Microbial communities, Cellular microbiology

## Abstract

Spatial organization within bacterial communities plays a critical role in mediating cell-cell interactions and determining microbial fitness. During infection, *Vibrio cholerae* undergoes dynamic restructuring of its spatial organization, forming monospecific aggregates thought to enhance survival in the face of the host immune system. However, the effectiveness of its primary weapon against other bacteria—its contact-dependent type VI secretion system (T6SS)—is severely limited in this aggregated form, hampering its ability to compete for space and resources. Here, we show that the presence of competing, co-resident bacteria alters *V. cholerae* aggregation by modulating toxin co-regulated pilus (TCP) expression through production of interspecies quorum sensing signal autoinducer-2 (AI-2). Using a zebrafish infection model, we found that this quorum sensing-controlled disaggregation enhances the efficacy of T6SS-mediated killing in vivo by promoting intermixing of *V. cholerae*, thereby increasing cell-cell contact with competitors. This modulation of aggregation has no impact on T6SS activity in vitro, highlighting the context-specific nature of these interactions. We developed a mathematical model to explore these dynamics and observed a fundamental trade-off between potency of *V. cholerae* T6SS and its sensitivity to the presence of competing bacterial species. Our findings reveal a core mechanism underlying *V. cholerae* colonization wherein it uses quorum sensing to dynamically balance between protective aggregation to survive host defenses and dispersed infiltrative intermixing to facilitate elimination of competitors.

## Abstract (web summary)

The pathogenic bacterium *Vibrio cholerae* forms aggregates within its host, which helps resisting host defenses. Here, Virgo et al. show that, upon sensing signals produced by competing bacteria, *V. cholerae* disperses its aggregates, facilitating interspecies mixing and the killing of the competing bacteria via the pathogen’s contact-dependent secretion system.

## Introduction

Bacteria growing in complex polymicrobial communities often adopt distinct patterns of spatial organization that both influence and are influenced by competitive and cooperative interactions^1–3^. Spatial constraints are particularly relevant for antagonistic contact-dependent interactions, such as the type VI secretion system (T6SS), because bacterial cell death only occurs at the interface between ‘prey’ cells and T6SS carrying ‘attackers’^4,5^. The T6SS is a widely conserved antagonistic weapon that injects toxic effector proteins directly into adjacent cells. In well-mixed multispecies communities, T6SS activity can lead to culling of sensitive populations^6,7^, but in lower density, non-motile, or mutually antagonistic systems, T6SS-mediated killing can result in self-segregation of bacterial species^3,8–10^. This effective separation of attacker and prey cells prevents further T6SS-mediated killing, presenting a significant constraint on T6SS efficiency. Considering that effective killing of prey cells can benefit bacteria in several ways, including space creation^10^, nutrient acquisition^11^, and gene scavenging^12^, bacteria relying upon contact-dependent systems have evolved strategies to overcome the self-limiting nature of contact-dependent weapons^10,13^.

In *Vibrio cholerae*, T6SS-mediated killing plays a critical role during infection. T6SS-mediated killing is active in the gut^14^ and provides a limited competitive advantage for colonization of specific anatomical locations^15^. There is also growing evidence that *V. cholerae* T6SS-mediated killing of resident gut bacteria leads to increased *V. cholerae* growth and pathogenicity, likely through modulation of host immune responses^16–18^ or even immune cell predation^19^.

The *V. cholerae* life cycle involves transitioning between the aquatic environment and human host^20^. During this process it switches between planktonic and multicellular forms^21^. Both forms exist in the aquatic environment, but biofilm-associated *V. cholerae* appear to be more infectious than the planktonic form after oral entry into the host gut^22^. Once bacteria reach the intestinal lumen, passage through mucosal layers to reach the epithelial surface requires motility and chemotaxis^23,24^, implying that *V. cholerae* transitions to a planktonic state during infection. At the epithelial surface, *V. cholerae* represses motility and forms surface microcolonies as it activates the rest of its virulence program^25,26^. In later stages of infection, virulence factors are eventually repressed and hemagglutinin/protease HapA is secreted, enabling *V. cholerae* to be shed back into the intestinal lumen and released into the environment as planktonic cells or multicellular aggregates^27,28^. Throughout this life cycle, environmental cues (e.g. temperature, pH and osmolarity^29^), in vivo signals (e.g. oxygen levels^30^, indole^31^, bile salts^32,33^), and bacterial quorum sensing signals^34^ regulate both *V. cholerae* virulence gene expression and multicellularity programming.

Both planktonic and multicellular aggregate states are important for disease progression and transmission with each conferring distinct context-dependent advantages. Multicellular aggregates and biofilm formation can increase antimicrobial tolerance^21^, provide protection from phagocytosis^19^, and enhance resistance to phage infection and bacterial antagonism^35^. By contrast, being separate motile cells allows for better penetration of host barriers^36^, competition escape^37^, and enhancement of contact-dependent antagonism by increasing population mixing^13^. As such, the in vivo aggregation dynamics of *V. cholerae* are particularly relevant for its T6SS-mediated antagonism.

T6SS activity has long been established to be functional only among bacterial communities growing on solid media^38^, requiring interspecies bacterial adhesion for liquid-phase killing to occur^39^. However, bacterial growth within an animal host represents a unique phase distinct from fully liquid or solid surface competition conditions frequently modeled in vitro^40^ with bacterial cell-cell interactions having significant differences in observed efficiency. For example, while T6SS-mediated antagonism can be observed in vivo, this activity is significantly less than bacterial populations of comparable density growing in vitro on solid media^18^. Conversely, other contact-dependent processes, such as DNA conjugation, are significantly more efficient in vivo than among comparable bacterial populations in vitro^15^.

Although the mammalian gut is a more natural in vivo model system for understanding *V. cholerae* infection, the presence of commensal gut microbes complicates disentanglement of specific interbacterial interactions. By contrast, the hindbrain ventricle (HBV) of zebrafish (*Danio rerio*) larvae, which has long been used as a model for enteric pathogen infection^41^, is a fully enclosed, naturally sterile compartment, allowing for study of defined mixtures of bacterial species in vivo independent of resident microbiota interactions^18,40^. Additionally, transparency of zebrafish larvae enables fluorescent visualization of these bacterial communities as they develop over time^40,42^, allowing for in depth contextualization of interbacterial dynamics within the host.

In this work, we sought to better understand T6SS killing dynamics of *V. cholerae* growing in vivo. While visualizing T6SS activity between *V. cholerae* and *Escherichia coli*, we observed that the presence of prey *E. coli* appeared to be modulating the *V. cholerae* transition between aggregates and planktonic individuals. We determined that *V. cholerae* aggregation under these conditions is mediated by toxin co-regulated pilus (TCP) and that autoinducer-2 (AI-2) produced by *E. coli* induces disaggregation. We further observed that TCP-dependent aggregation actively excludes cells lacking TCP, including *E. coli*, from cell aggregates, thereby limiting interspecies cell-cell contacts. When AI-2 biosynthesis is deleted from *E. coli*, *V. cholerae* do not undergo disaggregation, resulting in significantly less prey *E. coli* being killed by *V. cholerae* T6SS attack. This quorum sensing-controlled enhancement of T6SS activity demonstrates that *V. cholerae* can sense prey bacteria and modulate structural organization of the bacterial community to more effectively eliminate it. Notably, aggregation effects on T6SS-mediated killing were only present in vivo. In vitro competitions, on solid media or in liquid culture, were unaffected by the presence or absence of quorum sensing signals, hinting that the observed prey-hunting behavior of *V. cholerae* could be relevant during infection. Indeed, we found that several different resident bacteria species isolated from mammalian small intestine and feces are capable of inducing *V. cholerae* disaggregation like *E. coli*. We tested our hypothesis for feasibility using a mathematical model, which revealed a trade-off between the efficacy of T6SS killing and quorum sensing required for *V. cholerae* to successfully dominate the community. Ultimately, our findings show that *V. cholerae* TCP-mediated aggregation, typically thought to mediate host-pathogen interactions^43–45^, can also play an important role in mediating pathogen interaction with resident bacteria in the host.

## Results

### *V. cholerae* aggregation is reduced in the presence of *E. coli*

**Fig. 1:**
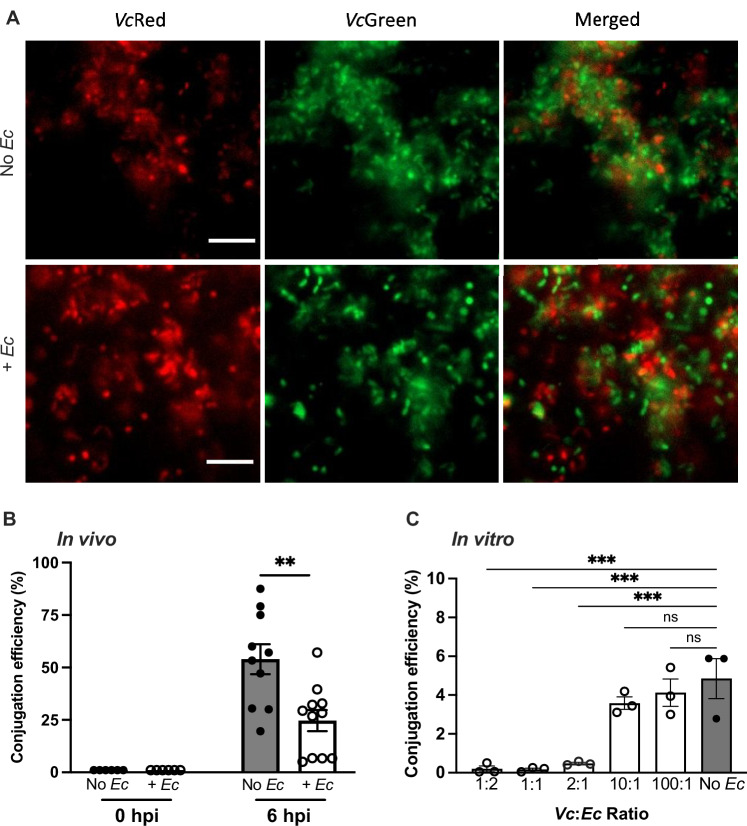
*V. cholerae* aggregation is reduced in the presence of *E. coli.* **A** Wild type AB larvae were co-injected in the hindbrain ventricle (HBV) with ~750 CFU *V. cholerae* constitutively expressing *sfCherry* (*Vc*Red) and *V. cholerae* constitutively expressing *mNeonGreen* (*Vc*Green) with no *E. coli* (top panel, No *Ec*) or with ~1500 CFU *E. coli* (no color) (bottom panel, + *Ec*). Larvae were imaged at 40x magnification. Representative image from single larvae at 6 h post infection shown. Single slice from a Z-stack taken. Scale bar, 10 μm. **B** Conjugation efficiency at 0- or 6- hours post infection (hpi) following injection of wild type AB larvae in the HBV with ~750 CFU *V. cholerae* wild type donor (Cm^S^, J13^+^) and recipient (Cm^R^) (No *Ec*) or ~750 CFU *V. cholerae* wild type donor (Cm^S^, J13^+^) and recipient (Cm^R^) and 1500 CFU *E. coli* (+ *Ec*). Data were pooled from three experiments, each with 3-4 larvae per time point (total n = 10 or 11). Each circle represents conjugation efficiency within a single larva. Significance was assessed using a two-sided unpaired t-test (No *Ec* vs + Ec at 6 hpi, *p* = 0.004). **C** Conjugation efficiency between *V. cholerae* wild type donor (Cm^S^, J13^+^), and recipient (Cm^R^) at 3 h post-mixing. *E. coli* was titrated in at ratios of 1:2, 1:1, 2:1, 10:1, 100:1 (total *V. cholerae* (*Vc*): *E. coli* (*Ec*)). An equal volume of LB was added in the instance where no *E. coli* was added (No *Ec*). Three biological replicates were performed for each condition. Significance was assessed using one-way ANOVA with Dunnett’s multiple comparisons test (adjusted *p*-values: 1:2 vs No *Ec*, *p* = 0.0002; 1:1 vs No *Ec*, *p* = 0.0002; 2:1 vs No *Ec*, *p* = 0.0004). ns not significant. For all panels, *V. cholerae* strain used is C6706 and bars indicate mean ± SEM. Source data are provided as a Source Data File.

In previous work, we used a zebrafish HBV model to study T6SS-mediated killing of *E. coli* by *V. cholerae*^18^. We found that the T6SS could not fully eliminate prey bacteria resulting in prolonged immune activation, presumably due to perpetual release of inflammatory cell debris resulting from continuous bacterial killing. To better understand structural organization underlying the inefficiency of T6SS-mediated bacterial antagonism, we used fluorescence microscopy to visualize *V. cholerae* populations inside the zebrafish HBV and made a striking observation: in the absence of *E. coli, V. cholerae* formed tight cellular aggregates, but when *E. coli* was present, a significant portion of *V. cholerae* cells were free swimming individuals. We co-injected into the zebrafish HBV a mixture of two isogenic *V. cholerae* strains constitutively expressing either *sfCherry* (*Vc*Red) or *mNeonGreen* (*Vc*Green), analogous to a similar two-color approach previously used to resolve in vivo localization dynamics in mice^25^. However, at 6 h post infection (hpi), we observed assemblages consisting of both red- and green- colored cells, indicating formation of non-clonal aggregates in vivo (Fig. 1A, Supplementary Movie S1), rather than clusters of cells derived from expansion of a single. Notably, when *E. coli* was co-injected alongside *V. cholerae* mixture, we observed substantially more individual free-floating *V. cholerae* cells, suggesting that presence of *E. coli* was either preventing or disrupting formation of *V. cholerae* aggregates (Fig. 1A, Supplementary Movie S2).

To quantify *V. cholerae* aggregation, we used a previously described genetic assay for cell-cell contact^15^. This assay exploits conjugation efficiency of a *Vibrio-*specific conjugative plasmid, P-factor^46^, as a proxy metric for cell-cell contact within a *V. cholerae* population. When donor and recipient cells aggregate with each other, there will be more cell-cell contacts and therefore more opportunities for plasmid transfer to occur. In our experiments, we used donor and recipient *V. cholerae* that were chloramphenicol sensitive (Cm^S^) and resistant (Cm^R^), respectively. The conjugative plasmid used is a variant of P-factor, J13, which confers carbenicillin resistance^15^. Conjugation efficiency was measured as the fraction of Cm^R^ recipient cells that are also carbenicillin resistant after mixing.

Larvae were co-infected with a 1:1 mixture of donor and recipient *V. cholerae* in the presence or absence of *E. coli*. Colony forming units (CFU) of donor, recipient, and transconjugant *V. cholerae* at 0- and 6- hpi were counted by homogenizing larvae and plating on media selective for each strain. In this case we observed that conjugation efficiency significantly decreased in the presence of *E. coli* (Fig. 1B), confirming a reduction in *V. cholerae* cell-cell interactions.

Having observed this reduction in conjugation efficiency in vivo, we next sought to determine whether this behavior could be reproduced outside the host. When *V. cholerae* was grown to exponential phase and concentrated to a cell density of at least OD_600_ = 1.0, ~8% of all recipients became transconjugants within 3 h of being mixed (Supplementary Fig. 1A). Adding *E. coli* into the cell mixture such that it made up less than 10% of the initial population had little to no effect on conjugation rates. However, when *E. coli* made up at least one third of the initial population, we observed a stark decrease in the amount of conjugation between *V. cholerae* donor and recipients, indicating a decrease in *V. cholerae* aggregation (Fig. 1C). We also confirmed that similar modulation of aggregation could be observed in other strains of *V. cholerae*, including the nontoxigenic environmental isolate *2740-80* which has a constitutively active T6SS^47^, as well as the epidemiologically relevant Haiti outbreak strain KW3^48^ (Supplementary Fig. 1B), highlighting that this observation is not a strain-specific effect.

### *V. cholerae* aggregation is mediated by TCP

To better understand how *E. coli* was modulating *V. cholerae* aggregation, we next sought to determine what was mediating *V. cholerae* cell-cell interactions. Several factors have previously been described to control multicellular community formation in *V. cholerae*, including T6SS^49^, the three type IV pili (T4P) found in *V. cholerae*: chitin-regulated competence pilus (ChiRP; formerly termed PilA)^49^, mannose-sensitive hemagglutinin (MSHA) pilus^26,50^, and toxin coregulated pilus (TCP)^51,52^; flagella^53^, lipopolysaccharide (LPS)^54^ and *Vibrio* exopolysaccharide (VPS)^55^. Specifically, we selected *V. cholerae* mutants with transposon (TnFGL3) disruptions in genes *vipA* (T6SS), *mshA* (MSHA)*, pilA* (ChiRP)*, tcpC* (TCP)*, flaA* (flagella)*, waaL* (LPS)*, vpsL* (VPS) and *vpsA* (VPS), from an established *V. cholerae* transposon mutant library^56^ and measured conjugation efficiency among these mutants in the presence or absence of *E. coli*.

**Fig. 2:**
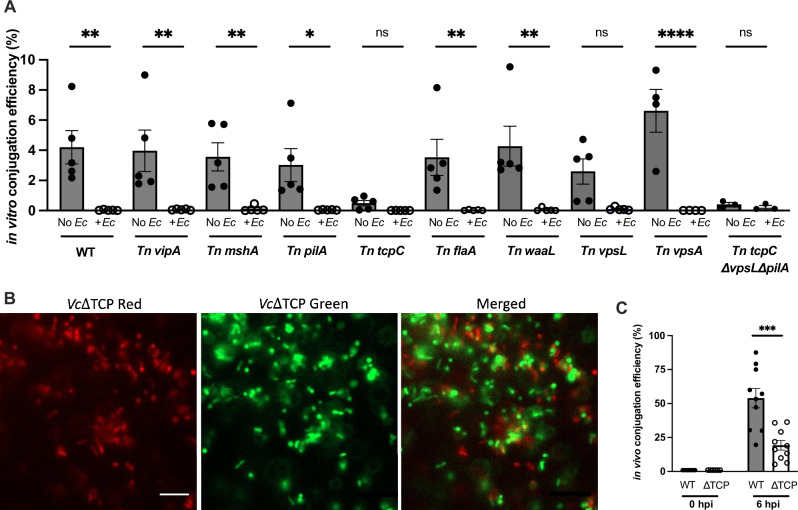
*V. cholerae* aggregation is mediated by TCP. **A** Conjugation efficiency between *V. cholerae* wild type (WT) or transposon mutant (*vipA*::TnFGL3*, mshA*::TnFGL3*, pilA*::TnFGL3*, tcpC*::TnFGL3, *flaA*::TnFGL3, *waaL*::TnFGL3, *vpsL*::TnFGL3, *vpsA*::TnFGL3, and triple mutant *tcpC::*TnFGL3∆*vpsL*∆*pilA*] donor (Cm^S^, J13^+^) and isogenic recipient (Cm^R^) at 3 h post-mixing. *E. coli* (+*Ec*) or an equivalent volume of LB (No *Ec*) was added at a 1:1 ratio (total *V. cholerae*: *E. coli*). Significance was assessed using a two-way ANOVA with a strain-by-condition interaction fitted to the data, followed by within-strain contrasts comparing the two conditions for each *V. cholerae* strain, with Holm correction applied across each multiple pairwise test (WT, *n* = 5, *p* = 0.0015; *vipA::Tn*, *n* = 5, *p* = 0.0030; *mshA::Tn*, *n* = 5, *p* = 0.0086; *pilA::Tn*, *n* = 5, *p* = 0.0251; *tcpC::Tn*, *n* = 5, *p* = 1.0000; *flaA::Tn*, *n* = 5, *p* = 0.0086; *waaL::Tn*, *n* = 5, *p* = 0.0014; *vpsL::Tn*, *n* = 5, *p* = 0.0633; *vpsA::Tn*, *n* = 4, *p* < 0.0001; *tcpC:Tn* ∆*vpsL* ∆*pilA*, *n* = 3, *p* = 1.0000). ns not significant. **B** Wild type AB larvae were injected in the hindbrain ventricle (HBV) with ~750 CFU *V. cholerae tcpC*::TnFGL3 (∆TCP) constitutively expressing *sfCherry* (*Vc*∆TCP Red) and ~750 CFU *V. cholerae* ∆TCP constitutively expressing *mNeonGreen* (*Vc*∆TCP Green). Larvae were imaged at 40× magnification. Representative image from single larvae at 6 hours post infection shown. Single slice from a Z-stack taken. Scale bar, 10 μm. **C** Conjugation efficiency at 0- or 6- hours post infection (hpi) following injection of wild type AB larvae in the HBV with ~750 CFU *V. cholerae* WT donor (Cm^S^, J13^+^) and recipient (Cm^R^) (WT) or ~750 CFU *V. cholerae* ∆TCP donor (Cm^S^, J13^+^) and recipient (Cm^R^) (∆TCP). Data were pooled from three independent experiments, each with 3–4 larvae per time point (*n* = 10). Each circle represents conjugation efficiency within a single larva. Significance was assessed using a two-sided unpaired t-test (WT vs ∆TCP at 6 hpi, *p* = 0.0008). For all panels, *V. cholerae* strain used is C6706 and bars indicate mean ± SEM. Source data are provided as a Source Data File.

Aside from the *tcpC* mutant (∆TCP), the presence of *E. coli* significantly reduced conjugation efficiency (Fig. 2A) for all other mutants. To confirm that the ∆TCP mutant was not intrinsically less capable of conjugation, we spotted the mutant onto solid media to force close association of bacteria even without active aggregation effects and observed no defect in conjugation efficiency (Supplementary Fig. 2). The low baseline conjugation efficiency for *V. cholerae* ∆TCP in the absence of *E. coli* suggests that TCP likely mediates aggregate formation under these conditions. We also deleted *pilA* and *vpsL* in the *tcpC::TnFGL3* strain to see if there were any synergistic effects, but these additional mutations did not reduce measured aggregation any further. When the mutant ∆TCP strain was injected into the HBV, we observed that *V. cholerae* cells did not form large aggregates by 6 hpi (Fig. 2B, Supplementary Movie S3). Moreover, conjugation efficiency in vivo was significantly lower among *V. cholerae* ∆TCP cells compared to wild type (WT) *V. cholerae* (Fig. 2C).

### AI-2 mediates *E. coli*-dependent disaggregation of *V. cholerae*

**Fig. 3:**
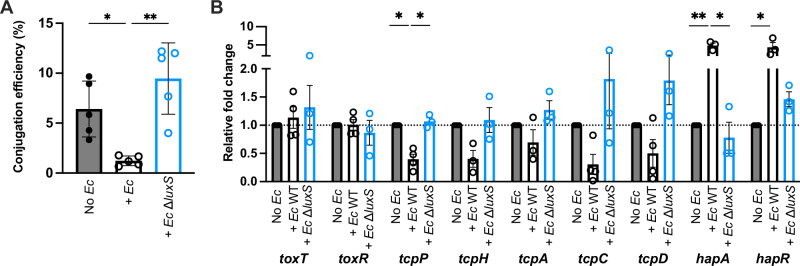
AI-2 mediates *E. coli*-dependent disaggregation of *V. cholerae.* **A** Conjugation efficiency between *V. cholerae* wild type (WT) donor (Cm^S^, J13^+^), and recipient (Cm^R^) at 3 hours post-mixing. *E. coli* WT (+*Ec*), *E. coli* ∆*luxS* (+*Ec* ∆*luxS*) or an equivalent volume of fresh LB (No *Ec*) was added at a 1:1 ratio (total *V. cholerae*: *E. coli*). Five biological replicates were performed for each condition. Significance was assessed using one-way ANOVA (No *Ec* vs +*Ec*, *p* = 0.027; +*Ec* vs +*Ec∆luxS*, *p* = 0.001; No *Ec vs* + *Ec∆luxS*, *p* = 0.25). **B** Fold change in the expression of *toxT, toxR, tcpP, tcpH, tcpA, tcpC, tcpD, hapA* and *hapR* in *V. cholerae* WT grown in the presence of *E. coli* WT (+*Ec* WT), *E. coli* ∆*luxS* (+*Ec* ∆*luxS*) or an equivalent volume of LB (No *Ec*) (1:1 ratio, total *V. cholerae*: *E. coli*). The relative gene expression was determined by normalization to *dnaB* as a housekeeping gene, and to *V. cholerae* grown without *E. coli* (No *Ec* condition) using the ∆∆Ct method. Data is representative of 3–4 biological replicates, each representing the average of two technical duplicates. Significance was tested using two-sided unpaired t-test on log_2_ transformed values (*n* = 3 for *toxT*, *toxR*, *tcpP*, *tcpC*, *tcpD*; *n* = 4 for *tcpH*, *tcpA*, *hapA*, *hapR*) (*tcpP* No *Ec* vs +*Ec*WT, *p* = 0.030; *tcpP* + *Ec*WT vs +*Ec∆luxS*, *p* = 0.023; *hapA* No *Ec* vs +*Ec*WT, *p* = 0.009; *hapA* + *Ec*WT vs +*Ec∆luxS*, *p* = 0.015; hapR No *Ec* vs +*Ec*WT, *p* = 0.047). For all panels, *V. cholerae* strain used is C6706 and bars indicate mean ± SEM. Source data are provided as a Source Data File.

Quorum sensing has been shown to modulate *V. cholerae* biofilm formation^34^, aggregation^57^ and can interfere with virulence gene expression^58^. The interspecies quorum sensing autoinducer-2 (AI-2) can drive biofilm dispersal in *V. cholerae*^34^, presumably through downregulation of TCP^59^ and increased expression of secreted protease *hapA*^60^. Given the threshold effect we observed for how much *E. coli* was needed to induce *V. cholerae* disaggregation (Fig. 1C), we hypothesized that interspecies quorum sensing was mediating this behavior. To test this hypothesis, we deleted *luxS* from *E. coli*, which encodes a key enzyme required for AI-2 synthesis^61^. This deletion increased *V. cholerae* conjugation to levels comparable to the *E. coli*-free condition (Fig. 3A). Furthermore, at a transcriptional level, elimination of *luxS* restored expression levels of TCP genes to those of the *E. coli*-free condition (Fig. 3B), suggesting that *V. cholerae* is detecting *E. coli* through its AI-2 production. To confirm the importance of AI-2, we constructed two mutant *E. coli* strains where genes involved in the uptake and utilization of AI-2, *lsrK* and *lsrR*, were deleted. In ∆*lsrK* mutants, AI-2 utilization is disrupted resulting in excess AI-2 accumulation in the supernatant, while in ∆*lsrR* mutants, AI-2 utilization genes are constitutively expressed resulting in continuous consumption of AI-2 independent of AI-2 accumulation^62^. While both mutants disrupt *V. cholerae* aggregation like WT *E. coli*, both require fewer cells to achieve the same effect (Supplementary Fig. 3A). This result is as expected for the ∆*lsrK* mutant which produces more AI-2. However, the ∆*lsrR* mutant also has an equivalent effect on *V. cholerae* aggregation, suggesting that *V. cholerae* might also respond to depletion of AI-2. Indeed, eliminating the ability for *V. cholerae* to detect AI-2 by deleting *luxQ*, a part of the AI-2 receptor for AI-2 resulted in *V. cholerae* aggregation becoming insensitive to *E. coli* with the strain remaining disaggregated at a baseline level (Supplementary Fig. 3B).

### TCP-mediated interactions exclude *E. coli* from aggregates and minimize antagonistic interspecies interactions

**Fig. 4:**
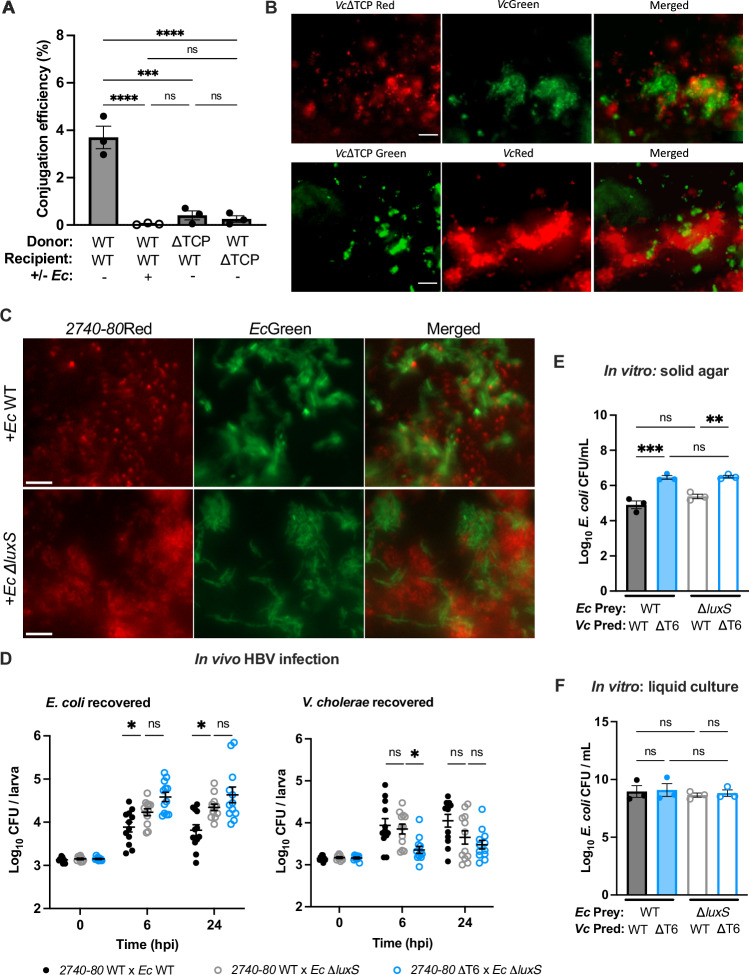
TCP mediated interactions exclude *E. coli* from aggregates and minimize antagonistic interspecies interactions. **A** Conjugation efficiency between wild type (WT) donor (Cm^S^, J13^+^) and ∆TCP recipient (Cm^R^) or ∆TCP donor (Cm^S^, J13^+^) and WT recipient (Cm^R^) *V. cholerae* at 3 hours post-mixing. *E. coli* (+*Ec*) or an equivalent volume of LB (-*Ec*) were added at a 1:1 ratio (total *V. cholerae* (*Vc*): *E. coli* (*Ec*)). *V. cholerae* strain is C6706. Three biological replicates were performed for each condition. Significance was assessed using one-way ANOVA (+*Ec* vs -*Ec* in WT donor/WT Recipient, *p* < 0.0001; WT Recipient vs ∆TCP Recipient with WT donor and -*Ec*, *p* < 0.0001; WT donor vs ∆TCP donor with WT Recipient and -*Ec*, *p* = 0.0001). **B** Wild type AB larvae were injected in the hindbrain ventricle (HBV) with ~750 CFU *V. cholerae* ∆TCP constitutively expressing *sfCherry* (*Vc*∆TCP Red) and ~750 CFU *V. cholerae* WT constitutively expressing *mNeonGreen* (*Vc*Green) (top panel) or ~750 CFU *V. cholerae* WT constitutively expressing *sfCherry* (*Vc*Red) and ~750 CFU *V. cholerae* ∆TCP constitutively expressing *mNeonGreen* (*Vc*∆TCP Green) (bottom panel). Larvae were imaged at 40× magnification. Representative image from single larvae at 6 h post infection (hpi) shown. *V. cholerae* strain is C6706. Single slice from a Z-stack taken. Scale bar, 10 μm. **C** Wild type AB larvae were injected in the HBV with ~1500 CFU *V. cholerae 2740-80* WT constitutively expressing *sfCherry* (*2740-80*Red) and ~1500 *E. coli* Δ*luxS* (top panel) or *E. coli* WT (bottom panel) constitutively *mNeonGreen* (*Ec*Green). Larvae were imaged at 40× magnification. Representative image from single larvae at 6 h post infection shown. Single slice from a Z-stack taken. Scale bar, 10 μm. **D** Enumeration of recovered *V. cholerae 2740-80* or *E. coli* at 0-, 6-, or 24- hpi from larvae infected in the HBV with *V. cholerae 2740-80* WT and *E. coli* WT (*2740-80* WT x *Ec* WT), *V. cholerae 2740-80* WT and *E. coli* Δ*luxS* (*2740-80* WT x *Ec* Δ*luxS*) or *V. cholerae 2740-80* ΔT6SS and *E. coli* Δ*luxS* (*2740-80* ΔT6 x *Ec* Δ*luxS*). Larvae were co-infected with ~1500 CFU of *V. cholerae* and *E. coli* each. Circles represent individual larvae pooled from 3 separate experiments, each with 3 larvae at 0 hpi (total *n* = 9) and 4 at 6 hpi and 24 hpi (total *n* = 12). Significance was assessed using one-way ANOVA on log_10_ transformed values (*E. coli* recovery for WT x *Ec* WT vs WT x *EcΔluxS* at 6 hpi, *p* = 0.028; *E. coli* recovery for WT x *Ec* WT vs WT x *EcΔluxS* at 24 hpi, *p* = 0.027; *V. cholerae* recovery for WT x *EcΔluxS* vs ΔT6SS x *EcΔluxS* at 6 hpi, *p* = 0.020). **E** Recovery of *E. coli* after in vitro competition on solid agar. *V. cholerae 2740-80* WT or ΔT6SS mutant (ΔT6) were mixed with *E. coli* WT (*Ec* WT) or Δ*luxS* (*Ec* Δ*luxS*) at a 5:1 ratio (*V. cholerae: E. coli*) and spotted on an agarose pad. Bacteria were recovered after 3 h at 28.5 °C. Three biological replicates were performed for each strain combination. Significance was assessed by one-way ANOVA on log_10_ transformed values (*Vc* WT vs ∆T6 with *Ec* WT, *p* = 0.0004; *Vc* WT vs ∆T6 with *Ec ∆luxS*, *p* = 0.0035). **F** Recovery of *E. coli* after in vitro competition in liquid culture. *V. cholerae 2740-80* WT or ΔT6SS mutant (ΔT6) cultures were mixed with *E. coli* WT or Δ*luxS* (*Ec* Δ*luxS*) at a 5:1 ratio (*V. cholerae: E. coli*). Bacteria were recovered after 3 h at 28.5 °C. Three biological replicates were performed for each strain combination. Significance was assessed by one-way ANOVA on log_10_ transformed values. For all panels, bars indicate mean ± SEM. ns not significant. Source data are provided as a Source Data File.

We next explored the role of TCP-mediated aggregation in T6SS-mediated antagonism. Although aggregation can facilitate contact-dependent interactions^49^, that same aggregation can also block interactions between cells if potential interaction partners are actively excluded from aggregates. In the case of TCP-mediated aggregation, *V. cholerae* mutants lacking TCP have been reported to be excluded from microcolonies of WT *V. cholerae*^52^. We reaffirmed this result using our conjugation-based assay, observing almost complete loss of conjugation among cells if either the donor or recipient lacked TCP (Fig. 4A). In zebrafish, ∆TCP mutants do not form mixed aggregates with WT (Fig. 4B, Supplementary Movie S4 and Supplementary Movie S5), but they also do not completely remain as individual planktonic cells, likely due to the in vivo environment having some solid-phase characteristics that enable non-aggregating bacteria to form microcolonies.

We next sought to confirm that *E. coli*, which does not have TCP, would be similarly excluded from TCP-mediated aggregates. Since we are ultimately interested in the effect on T6SS-mediated killing, for these experiments we used the *V. cholerae* strain *2740-80*, which exhibits the same aggregative behavior as C6706 (Supplementary Fig. 1B) but has a constitutively active T6SS. We mixed *2740-80* constitutively expressing *sfCherry* (*2740-80*Red) and WT or ∆*luxS E. coli* constitutively expressing *mNeonGreen* (*Ec*Green WT or *Ec*Green ∆*luxS*). When WT *E. coli* was mixed with *V. cholerae*, there were many dispersed individual *V. cholerae* cells and very few large clusters consisting of only *E. coli* with no interspersed *V. cholerae cells* (Fig. 4C, Supplementary Movie S6). By contrast, when *luxS E. coli* was mixed with *V. cholerae*, there were large clusters of only *E. coli or V. cholerae* (Fig. 4C, Supplementary Movie S7). By counting recovered CFU of each bacterial species from zebrafish, we could see that this intermixing was directly correlated with a reduction in *E. coli* growth (Fig. 4D). By 6 hpi, *E. coli* ∆*luxS* survived competition with T6SS + *V. cholerae* approximately 3-fold better than *E. coli* WT, and by 24 hours, the amount of recoverable ∆*luxS* mutant *E. coli* was even higher (comparable to competition with *V. cholerae* lacking a functional T6SS). The ∆*luxS* mutant *E. coli* does not fair worse than the WT when each is injected alone (Supplementary Fig. 4) indicating that *luxS* plays no role in *E. coli* colonization of the zebrafish.

The lower survival of WT *E. coli* by 6 hpi did not immediately result in a concomitant increase in *V. cholerae* growth, suggesting that during initial stages of colonization, the two species are not in direct competition for growth resources. However, by 24 hpi, when the maximum capacity of the system to support bacterial growth is reached, the *E. coli* population will have established itself as tight microcolonies (Fig. 4C) such that the inability of *V. cholerae* to intermix with ∆*luxS E. coli* limits its ability supplant it. Indeed, when we imaged in vivo mixtures of *E. coli* and ∆T6SS *V. cholerae* unable to antagonize the *E. coli*, the *V. cholerae* was more challenging to detect by microscopy, likely having been cleared by host defenses. However, if *V. cholerae* can prevent establishment of these pockets of *E. coli*, either by slowing their formation or by penetrating into them, *V. cholerae* is able to carve out a larger colonizable space for itself. Altogether, these results show that *V. cholerae*, upon sensing *E. coli*, will restructure its spatial organization to enable better competition with that *E. coli* population. As such, when these bacteria are mixed under non-dynamic conditions, (e.g. on solid agar in vitro), or when T6SS-mediated contact-dependent antagonism is ineffective (e.g. in liquid culture in vitro), being able to modulate its aggregation should confer no advantage to *V. cholerae*. Indeed, when we mixed *V. cholerae* and *E. coli* in vitro on solid agar or in liquid culture (Fig. 4E, F), there was no difference in *E. coli* survival between the WT and ∆*luxS* mutant.

### Multiple different resident gut species disrupt *V. cholerae* aggregation

**Fig. 5:**
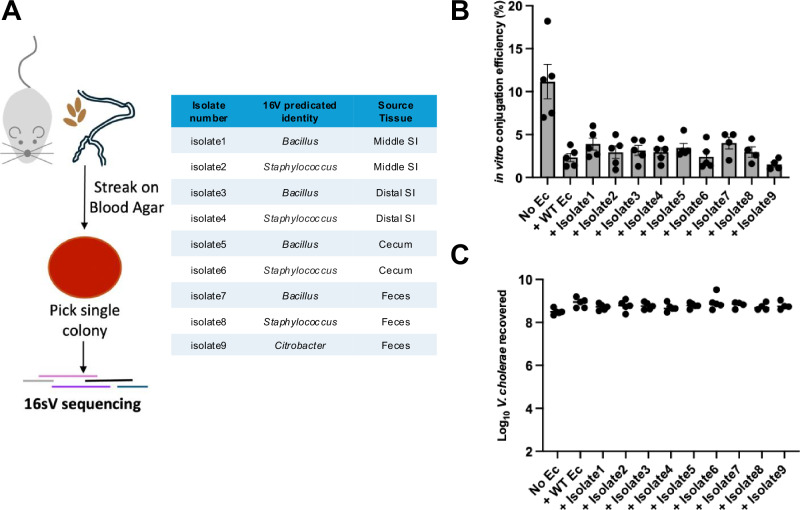
Multiple different resident gut species disrupt *V. cholerae* aggregation. **A** Samples were collected from the small intestine (SI), cecum, and feces of BALB/c mice, homogenized in PBS and streaked on blood agar plates to isolate resident bacteria. Individual colonies with distinct morphologies were selected. Sequencing of 16s variable regions (16sV) aided species identification. **B** Conjugation efficiency between donor *V. cholerae* wild type (WT) (Cm^S^, J13^+^) and recipient (Cm^R^) at 3 h post-mixing. *E. coli* (+ *Ec*), different mouse isolates (+Isolate) or an equivalent volume of LB (No *Ec*) were added at a 1:1 ratio (total *V. cholerae*: *E.coli*). *n* = 5 for all strain except isolates 7, 8, and 9, where *n* = 4. Significance was assessed using one-way ANOVA with Dunnett’s multiple comparisons test (adjusted *p*-values: *p* < 0.0001 for all strains vs No *Ec* control). **C** The total CFU of recovered *V. cholerae* from the conjugation efficiency assay for each isolate presented in (**B**). Bars indicate mean ± SEM. Source data are provided as a Source Data File.

Since the benefit of aggregation modulation on interbacterial competition was only observed in vivo, we next wondered whether these dynamics could be relevant during interactions with natural microbiota populations. The LuxS/AI-2 biosynthesis pathway is shared by a wide range of both Gram-positive and Gram-negative species^63^, including bacteria residing in the mammalian gut that *V. cholerae* might interact with during infection. To confirm whether such bacteria can trigger *V. cholerae* disaggregation, we isolated several bacteria species from the intestine and feces of an adult mouse (Fig. 5A). Sequencing of the 16S rRNA gene indicated that these isolates belonged to the genera *Staphylococcus*, *Bacillus, and Citrobacter*, all of which have been reported to produce AI-2^64–66^. Each of these isolates promoted disaggregation of *V. cholerae* in our conjugation-based assay (Fig. 5B). We confirmed that there was no change in the growth rates of *V. cholerae* during the conjugation assay (Fig. 5C), therefore loss in measured conjugation efficiency was likely not due to antibacterial antagonism against *V. cholerae*. To further explore the role of AI-2, we used an AI-2 biosensor strain^67^ to determine the relative amount of AI-2 produced by each of these isolates (Supplementary Fig. 5). As expected, all but one of these strains exhibited clear AI-2 production. Interestingly, one of the fecal isolates produced very little if any detectable AI-2, despite being able to modulate *V. cholerae* aggregation, suggesting that AI-2 may not be the only intercellular signal in natural microbiota that *V. cholerae* responds to.

### *V. cholerae* aggregation contributes to colonization of the zebrafish HBV

**Fig. 6:**
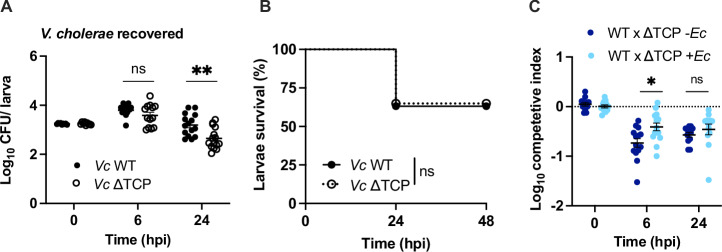
*V. cholerae* aggregation contributes to colonization of the zebrafish HBV. **A** Enumeration of recovered *V. cholerae* at 0-, 6-, or 24- hours post infection (hpi) from larvae infected with ~2000 CFU of wild type (*Vc* WT) or ΔTCP (*Vc* ΔTCP) *V. cholerae*. Circles represent individual larvae. Data were pooled from three independent experiments, each with 3–5 larvae per time point: 0 hpi (*n* = 9), 6 hpi (*n* = 11–13), 24 hpi (*n* = 15). Significance was assessed using two-sided unpaired t-test on log_10_ transformed values (WT vs ∆TCP at 24 hpi, *p* = 0.002). **B** Survival curves of larvae infected with ~2000 CFU of wild type (*Vc* WT) or ΔTCP (*Vc* ΔTCP) *V. cholerae*. Data were pooled from three independent experiments, each with 17–20 larvae. Significance was assessed using log-rank Mantel–Cox test. **C** Competitive index at 0-, 6- or 24 hpi following injection of wild type AB larvae in the HBV with a 1:1 mixture of *V. cholerae* WT and ∆TCP (~750 CFU each) (*Vc* WT x *Vc* ∆TCP *-Ec*) or a 1:1:2 mixture of *V. cholerae* WT and ∆TCP (~750 CFU each) and *E. coli* (~1500 CFU) (*Vc* WT x *Vc* ∆TCP +*Ec*). Data were pooled from four independent experiments, each with 3-4 larvae per time point: 0 hpi (*n* = 12), 6 hpi (*n* = 14–15), 24 hpi (*n* = 12). The competitive index was calculated by dividing the ratio of *V. cholerae* WT to *V. cholerae* ∆TCP by the input ratio. Significance was assessed using two-sided unpaired *t*-test (−*Ec* vs +*Ec* at 6hpi, *p* = 0.010; −*Ec* vs +*Ec* at 24hpi, *p* = 0.055). For all panels, *V. cholerae* strain is C6706 and bars indicate mean ± SEM. ns not significant. Source data are provided as a Source Data File.

In mammalian models, TCP mutants exhibit a colonization defect compared to WT^52^. In the zebrafish HBV, we observe a similar effect with the *V. cholerae* ∆TCP mutant having a reduced bacterial load compared to WT by 24 hpi in the absence of competing *E. coli* (Fig. 6A), though this difference in bacterial growth did not impact overall larvae survival (Fig. 6B). Given that *E. coli* modulates *V. cholerae* TCP, we wondered whether adding *E. coli* would eliminate the benefit conferred by TCP. We injected mixtures of WT and ∆TCP *V. cholerae* into the zebrafish HBV in the presence or absence of *E. coli* and determined the competitive index between WT and ∆TCP mutant. As expected, in the absence of *E. coli*, WT *V. cholerae* had an approximately 10-fold advantage over the ∆TCP mutant (Fig. 6C). Including *E. coli* in the mixture only partially abrogated the advantage of having TCP, suggesting that, although some disaggregation of WT *V. cholerae* is occurring, enough *V. cholerae* remains aggregated such that the advantage of having TCP is maintained. This interpretation is consistent with our microscopy results showing aggregated *V. cholerae* in the presence of *E. coli* (Fig. 1A). Additionally, when we consider raw cell counts, we could see that the effect of *E. coli* on the competitive index is due to a reduction in colonization by WT (Supplementary Fig. 6) with no corresponding change in the amount of ∆TCP mutant, suggesting that disaggregated *V. cholerae* do not occupy the same colonization niche as aggregated bacteria.

### Modeling aggregation and T6SS dynamics

To confirm that these mechanistic interpretations can, in principle, manifest our observations, we derived a modified Lotka-Volterra competition model^68^ based on a set of simple assumptions. In the model, we consider three interacting populations: *E. coli*, aggregated *V. cholerae*, and planktonic *V. cholerae*. Aggregated and planktonic *V. cholerae* are treated as distinct interconverting populations, with the first order rate constant for disaggregation assumed to increase linearly with *E. coli* concentration (with gradient Q). Thus, the ratio of *V. cholerae* in its aggregated versus planktonic form is determined by the amount of *E. coli* present (Fig. 4D). We assume that all populations compete for finite space. The T6SS-mediated inhibitory effect of planktonic *V. cholerae* on *E. coli* is represented by an additional negative interaction (α). Given that the ∆TCP mutant of *V. cholerae* does not reach as high densities as the WT (Fig. 6A, Supplementary Fig. 6), our model allocates aggregated *V. cholerae* a higher carrying capacity than planktonic *V. cholerae*. We explored the interaction between efficacy of killing and sensitivity of the quorum sensing effect by varying the parameter α and Q. A detailed description of the model is included in Supplementary File S1.

**Fig. 7:**
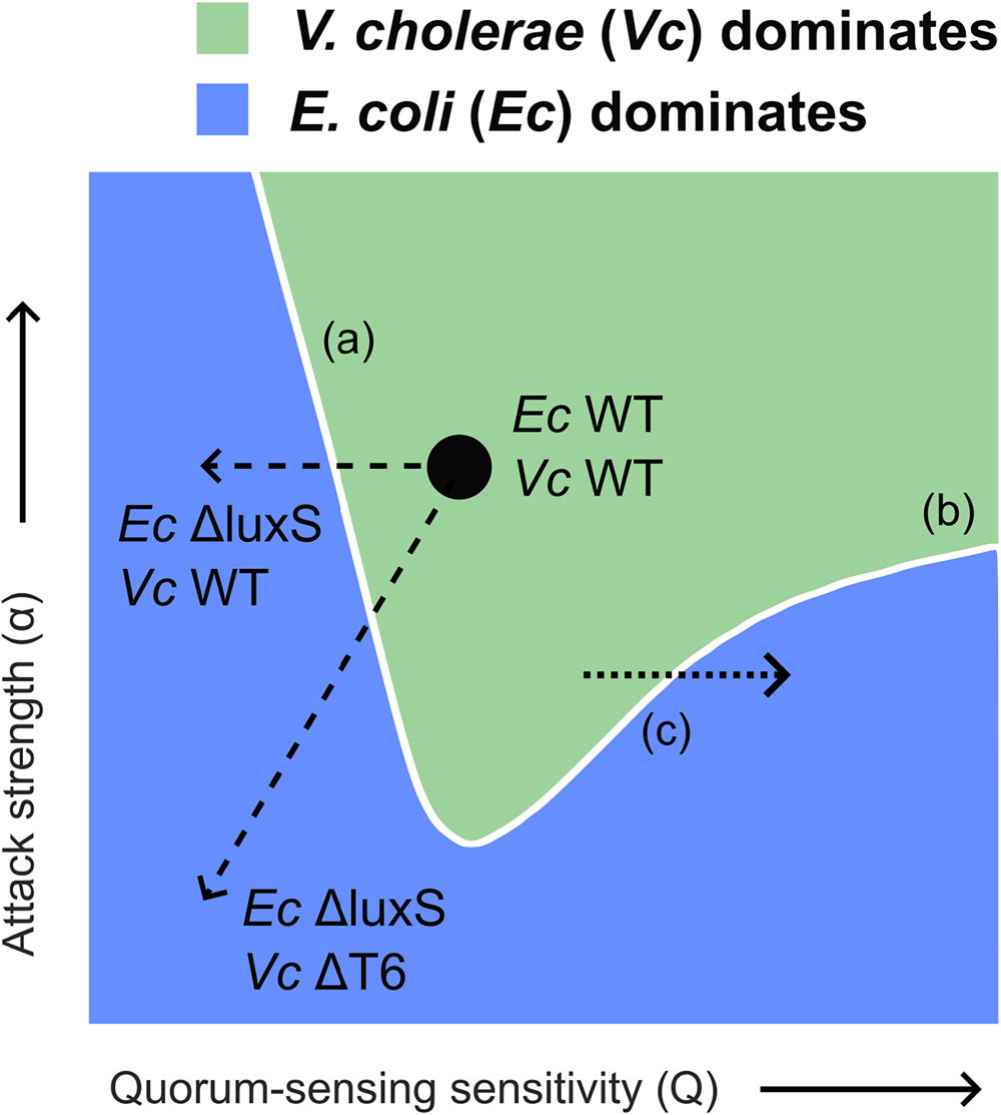
Results of simulated competition between *V. cholerae* and *E. coli.* Simulations based on a modified Lotka-Volterra competition model run to steady state results in competitive exclusion of either *V. cholerae* or *E. coli*. The parameter space shown illustrates how the efficacy of *V. cholerae* T6SS attack (y-axis) and *V. cholerae* responsiveness to the presence of *E. coli* (x-axis; i.e. the rate at which aggregates disaggregate) impact the outcome of the competition. The black circle represents a parameter set representative of competition between wild types (WT), while the labeled arrows indicate changes in parameters representative of competition between WT and mutant strains. Labels a, b, and c highlight distinct regimes discussed in the text. Model equations, code and parameter values used for the simulation are detailed in Supplementary File S1.

These simulations result in competitive exclusion of either *V. cholerae* or *E. coli*, depending on the value of α and Q (Fig. 7 and Supplementary Fig. 7). Although complete eradication is not realistic in a spatially heterogenous environment such as our experimental system, and the equilibrium state should not therefore be interpreted literally, our model highlights fundamental trade-offs between efficacy of the planktonic *V. cholerae* attack and strength of the quorum sensing response. From the model, two clear regimes emerge. In the first regime, when sensitivity of aggregated *V. cholerae* to quorum sensing signals is relatively low, there is a trade-off between quorum sensing sensitivity and T6SS efficacy. The more efficiently *V. cholerae* can respond to quorum sensing signals, the less potent its T6SS needs to be for *V. cholerae* to outcompete *E. coli* (labeled (a) in Fig. 7). In the second regime, when quorum sensing response of *V. cholerae* is strong, *V. cholerae* is permanently in its planktonic form, and only the efficacy of the T6SS dictates whether *V. cholerae* or *E. coli* outcompetes the other (labeled (b) in Fig. 7). Owing to the slight decrease in carrying capacity of planktonic *V. cholerae* versus aggregated *V. cholerae*, the model also predicts that under some conditions an increase in quorum sensing sensitivity can disfavor *V. cholerae*, and lead to a reduction in its ability to outcompete *E. coli* (labeled (c) in Fig. 7). This prediction may be consistent with observations of AI-2 producing bacteria being able to reduce *V. cholerae* colonization^69^.

## Discussion

**Fig. 8:**
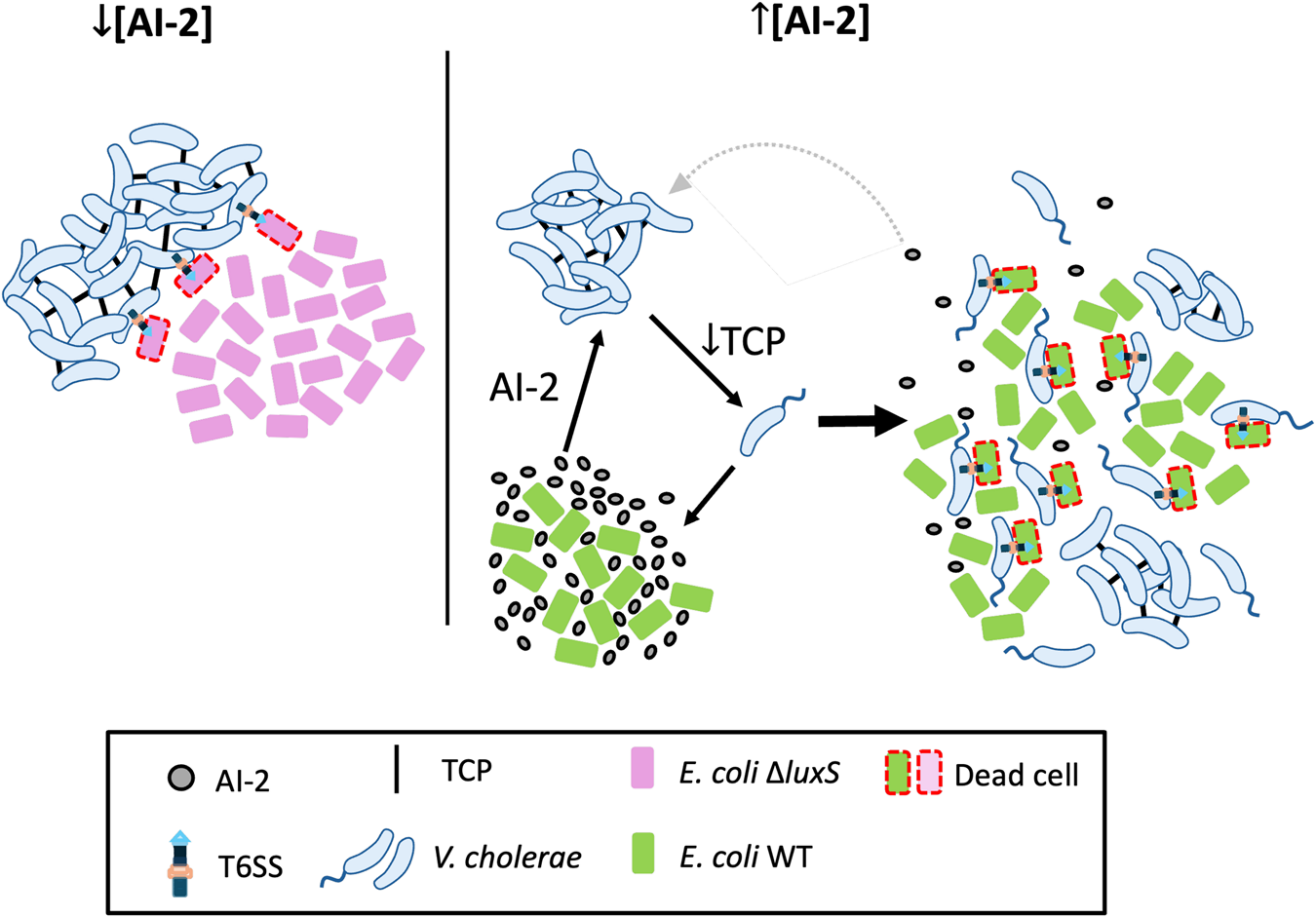
*V. cholerae* modulates its spatial organization in response to competing bacteria. When the concentration of AI-2 is low (*E. coli* ∆*luxS*) *V. cholerae* cells arrange into clusters of TCP-mediated aggregates. Inter-species contacts are limited to the *V. cholerae* aggregate boundary. When AI-2 concentration increases (*E. coli* WT), *V. cholerae* cells at least partially disassemble their TCP-mediated aggregates, enabling free-floating individual cells to invade clusters of competitor cells. This spatial reorganization increases interspecies cell-cell contacts enabling *V. cholerae* to more effectively ‘hunt down’ and target competing bacteria via its T6SS.

Contact-dependent modes of inter-bacterial antagonism are highly dependent on the spatial arrangement of bacterial cells for their ability to shape overall population dynamics. Here, we show that *V. cholerae* can modulate its spatial organization in response to competing bacteria, to enable more effective interbacterial antagonism in vivo. In response to interspecies quorum sensing signal AI-2, *V. cholerae* at least partially disassembles its TCP-mediated aggregates, enabling free-floating individual cells to penetrate into clusters of competitor *E. coli* cells. This spatial reorganization increases interspecies cell-cell contacts enabling the *V. cholerae* T6SS to more effectively restrict *E. coli* growth (Fig. 8). Notably, aggregation modulation control of T6SS activity only functions in the hybrid liquid/solid in vivo environment. Although *V. cholerae* does disaggregate in response to *E. coli* in liquid cultures in vitro, the absence of a solid surface support means that aggregation dynamics do not translate into changes in cell-cell contact conducive for T6SS-mediated killing. By contrast, bacteria deposited on solid media in vitro do not have the freedom to engage in dynamic spatial rearrangement.

General modulation of bacterial aggregation is a common feature of many bacterial community dynamics with several distinct examples having been reported recently. For example, *Aeromonas* species in the zebrafish gut can induce fragmentation of *Enterobacter* aggregates to alter its spatial organization and lower its abundance and growth rate^42^. *Streptococcus* species secrete a protease that cleaves a surface adhesin of *Staphylococcus*, disrupting its biofilms^70^. There are even examples of inter-domain interactions modulating community level aggregation and competition. The algae *Chlamydomonas reinhardtii* can inhibit *E. coli* aggregate formation, leaving bacteria more vulnerable to predation by other unicellular eukaryotes^71^. Fungal hyphae can drive *V. cholerae* dispersal through their associated water films^72^.

In the case of *V. cholerae*, we demonstrated how TCP-mediated aggregation can both promote and inhibit different forms of bacterial cell-cell interaction. Among aggregated populations, intra-*Vibrio* horizontal gene transfer is enhanced, as the extensive close contact between cells facilitates DNA conjugation. Meanwhile, aggregation can simultaneously sequester *V. cholerae* away from the surrounding environment, limiting its ability to antagonize and supplant competing bacterial populations. These results represent a novel aspect of TCP dynamics during infection. Classically, TCP pili enable microcolony formation to promote survival in the intestine^52^. Even in the zebrafish HBV, we observe that TCP enhances in vivo survival, presumably through providing protection from immune cells recruited to the infection site^73^. However, as the infection progresses and bacterial cell density increases, the benefit gained by aggregating together is negated by an inability to effectively compete with other co-resident bacteria. Given that TCP-mediated aggregation is controlled by interspecies quorum sensing, limited aggregate dispersal during infection likely represents a natural adaptation by *V. cholerae* to facilitate its invasion into resident microbiota populations. However, as our modeling of the system shows, aggregation modulation is potentially a double-edged sword in that complete loss of TCP results in clearance by the host. In the case of *E. coli*, which is sensitive to being killed by the T6SS of *V. cholerae*, enhancement of T6SS activity due to disaggregation effectively acts as a negative feedback limiter on production of quorum sensing signals. In agreement, a gnotobiotic mouse co-infection study has shown that *Blautia obeum*, which as a Gram-positive likely cannot be killed by *V. cholerae* T6SS, restricts *V. cholerae* colonization by producing AI-2 to repress *V. cholerae* virulence factor expression^69,74^. Ultimately, the emergent balance between aggregated and planktonic states will depend on species composition of the specific anatomical niche, which in turn will depend on quorum sensing signals^75^.

Beyond the gut, AI-2 has also been shown to influence microbial composition among reef-building coral, causing an increase in abundance of opportunistic pathogens, including *Vibrio* spp., and driving dysbiosis of coral-associated microorganisms^76^. Within these environments, *Vibrio* does not have access to host-derived nutrients^77^ and may instead rely upon nutrient acquisition from lysate of nearby bacteria that have been killed by *Vibrio* T6SS^11^. However, T6SS-mediated predation becomes less effective as a nutrient-scavenging mechanism as the proportion of prey cells decrease^78^. Therefore, being able to detect the proportion of ‘self’ versus ‘non-self’ in the microbial community, and respond accordingly, would be beneficial. When the number of non-self bacteria is above a certain threshold, *V. cholerae* can enter ‘hunter mode’, but as these bacteria are lysed, their numbers decrease and the need for competition or predation is no longer detected, *V. cholerae* can return to its aggregated state.

Spatial constraints can limit success of contact-dependent antagonism within a bacterial population^1,37,72,79,80^. For example, differential T4P expression among predator and prey cells in a bacterial population can lead to spatial segregation of piliated and non-piliated cells, limiting T6SS-mediated antagonism^37,79^. In this way, T4P-mediated exclusion of cell populations is a defense mechanism against contact-mediated attacks, while factors which promote the number of new contacts or the degree of intermixing will greatly impact the success of contact-dependent competitions e.g. predator-prey aggregation^81^ or predator motility^13^. Here, we observed how *V. cholerae* promotes aggregation between piliated cells, excluding non-piliated cells. Given that non-kin cell-cell contact may invite contact-dependent competition^49^ or allow cheaters to be introduced into the bacterial community, decreasing population fitness^82^, exclusion of non-piliated cells offers a way of protecting closely related individuals through aggregation and spatial segregation while excluding non-self-bacteria. We also observed that, on sensing *E. coli* via AI-2, *V. cholerae* can reorganize its population structure, thereby increasing the number of new contacts needed for successful T6SS-mediated competition.

Our study also highlights the strength of using the zebrafish HBV as a platform to simultaneously explore different types of bacterial cell-cell interactions within multi-species communities^18,40^. Introducing defined microbial communities into the HBV allowed us to uncover the interplay between spatial organization of the bacterial community and the bacterial cell-cell interactions active within it. This model will likely be transformative for future studies looking to further disentangle the interplay between bacterial interaction mechanisms and host factors, especially in situations where spatial geometry is a prevailing factor.

## Methods

### Bacterial strains and growth conditions

A detailed strain list can be found in Supplementary Table S1. *V. cholerae* genes *vc0729* and *vc1520* are neutral genes based on published transposon mutagenesis experiments^14,83^. *V. cholerae* and *E. coli* strains were cultured in Lennox formulation of LB (5 g/L NaCl) for liquid culture and on LB agar for stationary. Isolates from mouse fecal samples were isolated on blood agar.

Liquid and agar cultures were grown at 37 °C unless otherwise specified. Where needed, antibiotics were added at the following concentrations: kanamycin (50 μg/ml), streptomycin (50 μg/ml), carbenicillin (100 μg/ml), chloramphenicol (*V. cholerae* 3 µg/mL, *E. coli* 15 µg/mL). Diaminopimelic acid (DAP) was used at 0.3 mM for growth of MFD*pir E. coli*.

### Genetic manipulation in *E. coli*

To disrupt *luxS* in *E. coli, λ*red recombination was used^84,85^. Primers luxS_F and luxS_R were used to PCR amplify the kanamycin resistance (Kan^R^) cassette, flanked by FRT-sites from pKD4 with homology regions to *luxS*. The resulting PCR product was transformed in *E. coli* DH10β carrying the pKD46 plasmid to express the λ-Red genes. The success of chromosomal disruption of *luxS* was confirmed by PCR and sequencing using external primers, luxSconf_F and luxSconf_R (Supplementary Table S2). *E. coli* $\Delta$*luxS* strains were then transformed with pBAD33-pTAC-mNeonGreen.

### Construction of *V. cholerae* strains

To generate strains of *V. cholerae* carrying desired plasmids, plasmids were first transformed into electrocompetent *E. coli* MFD*pir* cells. These cells were then co-incubated with *V. cholerae* on LB agar for 1 h at 37 °C. Transconjugants were then isolated by selective plating on appropriate antibiotic and, where appropriate, confirmed for fluorescence.

### In vitro conjugation efficiency assays

For in vitro conjugation assays, 30 μl overnight (O/N) cultures of donor and recipient *V. cholerae* were sub-cultured in 4 mL fresh LB and grown to OD_600nm_ 0.4 (exponential phase) or OD_600nm_ 1 (late-log phase) as specified. Cells were centrifuged at 4000 × *g* for 5 min and pellets washed in LB, and resuspended to OD_600nm_ 10 (high density), 1 (medium density) or, 0.1 (low density), as specified. 30 μl O/N cultures of *E. coli* DH10β pBAD33-pTACmNeonGreen and mouse fecal or intestinal isolates were sub-cultured in 4 mL fresh LB and grown to OD_600nm_ 0.4 (exponential phase). Cells were centrifuged at 4000 × *g* for 5 min and pellets washed in LB, correcting the OD_600nm_ to 2 (unless otherwise specified). Donor and recipient cells were mixed at a 1:1 ratio in 1.5 mL Eppendorf’s. Additional LB, *E. coli* or mouse intestinal or fecal bacterial isolates, were added at an equal volume. For solid phase conjugation assays, 5 μl of donor and recipient bacteria were spotted onto warmed, dry agar plates.

For all conjugation assays, bacteria were incubated at 28.5 °C in a static incubator and CFUs were done after 0-, 2-, or 3- hrs post mixing, as specified in the text. To recover bacteria from solid phase conjugation assays, the spot was exercised and vortexed in 1000 μl LB. Selective media allowed distinguishment of donor, recipient and transconjugant bacteria. Conjugation efficiency was defined as the number of transconjugants divided by total number of recipients. A minimum of three replicates were performed for each group with each replicate being performed in technical duplicate. Colonies were counted after ~16 h incubation at 37 °C.

### In vitro T6SS competition assay

T6SS killing assays were performed following standard protocols^18,86^. Overnight cultures were diluted 100-fold in fresh media and grown to mid-log phase. Cells were pelleted, washed in fresh media, and resuspend to OD_600_ ≈ 10. Killer and prey cells were mixed at a 5:1 ratio (*V*. *cholerae* to *E*. *coli*) and 5 μL of the competition mix was spotted onto LB agar plates (solid media competitions) or 100 μl of bacterial liquid cultures were mixed in 1.5 mL tubes (liquid phase competitions) and incubated for 3 hrs at 28.5 °C. Competitions were then collected, serially diluted, and spotted on agar selecting for each strain to count surviving CFU. Experiments were performed in triplicate with each replicate being performed in technical duplicate.

### RNA extraction, cDNA synthesis and qRT-PCR

Bacteria cultures were grown to OD_600nm_ 0.4 (exponential phase) before centrifugation at 4000 × *g* for 5 min. The pellets were then washed using LB, correcting the OD_600nm_ to 1. *V. cholerae* was mixed at a 1:1 ratio with *E. coli* DH10β or LB. Bacteria were incubated at 28.5 °C in a static incubator for 3 hrs. An amount of culture corresponding to 1 × 10^9^ bacterial cells was pelleted by centrifugation at 5000 × *g* at 4 °C for 10 min, the supernatant was removed, and the pellet was kept at -80 °C overnight. RNA was then extracted with the RNeasy Mini kit (Qiagen cat no. 74104) as per manufacturer’s instructions. Eluted RNA was tested for quality and concentration using a nanodrop (DeNovix DS-11). 1000 ng of RNA was then converted to cDNA using the reverse-transcribed using QuantiTect Reverse Transcription kit (Qiagen cat no 205311) according to manufacturer’s instructions. Template cDNA was subjected to quantitative reverse transcription PCR (qRT-PCR) using 7500 Fast Real-Time PCR System machine and SybrGreen Mastermix (Applied Biosystems cat no. 10187094), with samples run in technical duplicates. Primers used can be found in Supplementary Table S2. The comparative Ct method was used for gene expression quantification and *dnaB* was used as the housekeeping gene.

### Ethics statement

Animal experiments were performed according to the Animals (Scientific Procedures) Act 1986 and approved by the Home Office (Project licenses: PP5900632). Each project license was reviewed and approved by the Animal Welfare and Ethical Review Body (AWERB) at LSHTM. All experiments were conducted up to 5 days post fertilization (dpf).

### Zebrafish husbandry

Embryos were obtained from naturally spawning zebrafish and maintained at 28.5 °C in 0.5× E3 medium supplemented with 0.3 g/mL methylene blue^87^. For injections, larvae at 3 dpf were anesthetized with 200 μg/mL tricaine in embryo medium and placed on a 1% agarose (Bioline) pad. WT-AB zebrafish were used for all in vivo experiments.

### Injection inoculum preparation

Infection methods were adapted from previous protocols^18,88–91^. For infection inoculum preparation, 400 µL of overnight cultures were sub-cultured in 20 mL fresh media supplemented with appropriate antibiotics and grown to OD_600nm_ 0.4–0.5. Bacteria were then harvested by centrifugation (4000 × *g*, 5 min), washed in 1 mL phosphate-buffered saline (PBS; Sigma Aldrich) to remove residual media, and pelleted again (1 min, 6000 × *g*) before resuspending in 300 µL 4% polyvinyl-pyrrolidone (PVP; Sigma-Aldrich). The desired inoculum concentration was achieved by measuring the OD_600nm_ of bacteria and correcting to desired OD_600nm_ in PVP and 0.5% phenol red dye (Sigma Aldrich). Addition of phenol red dye to injection inoculum allows injections to be visualized by optical microscopy. For co-infections, bacterial suspensions were mixed at the appropriate ratio immediately before injection.

### Zebrafish injections

Microinjections were performed in the hindbrain ventricle (HBV) site. 5 μL of suspension inoculum was loaded into the glass capillary needle and injected into a single larva. Injected embryos were then transferred into individual wells containing embryo medium and incubated at 28.5 °C.

### Quantification of bacterial burden

To determine injection inoculum or bacterial burden at later timepoints, larvae were mechanically homogenized in 200 μL of Lysis Buffer (PBS 0.1% TritonTM X-100) at 0 hours post infection (hpi) for injection inoculum and at 6- and 24- hpi for bacterial burden quantifications, as specified in the text. For each time point, 3–4 different larvae were selected at random as representatives for the infected population. Homogenates were serially diluted in PBS and plated onto selective plates. Colonies were counted manually following overnight incubation at 37 °C. Only larvae having survived infection were included in analyses.

### Survival assays

For survival assays, larvae were maintained in pairs in 24-well plates at 28.5 °C and visualized using a Leica KL300 LED microscope at 24- and 48- hpi to check survival. Larvae failing to produce a heartbeat within a 30 s period or in which bacteria had compromised the HBV were considered nonviable.

### Zebrafish microscopy and image analysis

To minimize autofluorescence during live imaging, embryo medium was switched to media not supplemented with methylene blue solution. To block pigmentation and improve optical transparency of larvae, embryo medium was supplemented with 0.003% 1- phenyl-2-thiourea (PTU) from 24 hpi^92^. For confocal microscopy, larvae were maintained in glass-bottom 35 mM dishes and imaged using a widefield microscope (Zeiss) with 40× dry objective. Live anaesthetized larvae were immobilized in 1% TopVision Low Melting Point Agarose in embryo medium (no methylene blue) supplemented with tricaine (200 μg/mL) and positioned yolk-sack up. Multiple position Z-stacks of the HBV were acquired with imaging at defined time points, as specified in the text^93^. Imaging was performed at 28.5 °C. Image files were processed using ImageJ/FIJI software. Microscopy images represent single slices of a Z-stack image series.

### AI-2 biosensor microscopy and image analysis

The test strain and the AI-2 biosensor strain, *E. coli ΔluxS* (*attB*::PlsrA-*yfp*)^67^, were grown overnight in 2 ml Lennox LB medium at 37 °C with shaking (200 rpm). The overnight cultures were subcultured into fresh LB and adjusted to OD₆₀₀  =  0.004 and mixed at a 1:1 ratio in a final volume of 2 ml, followed by incubation at 37 °C with shaking (200 rpm) for 16 h. Co-cultures were diluted into fresh LB, concentrated to OD₆₀₀  =  2.0, and spotted onto 1.5% (w/v) agarose pads prepared with M9 medium supplemented with 0.4% (w/v) casamino acids and imaged by fluorescence microscopy.

The fluorescence images were captured using a Nikon ECLIPSE Ti2 inverted microscope with a CoolLED pE4000 illuminator, Zyla 4.2 Megapixel Camera, and NIS-elements software. The microscopy images were analyzed using the MicrobeJ plug-in^94^ for Fiji^95^.

### Isolation of resident bacteria from adult mouse fecal and intestinal samples

Fecal and intestinal samples from adult BALB/c mice were collected, single pellets resuspended in 1 mL PBS and plated on Blood agar plates. Individual colonies with distinct morphology were selected and re-streaked on LB-agar. To determine strains of bacteria isolated from mouse feces, colony PCR with Q5 was performed using V6V9F and V6V9R, V1V2F and V1V2R and V3F and V3R primers (Supplementary Table S2) to amplify different 16 s variable regions. PCR products were purified and sent for sequencing. Species identification was achieved by BLAST.

### Quantification and statistical analysis

Statistical tests were performed using GraphPad Prism v9.4.1 software. Data are represented as the mean ± standard errors of the mean (SEM) from at least three independent biological replicates. For differences in survival curves, the log-rank (Mantel–Cox) test was used. Data from bacterial burden and gene expression levels were log_10_- or log_2_- transformed respectively. Pairwise comparisons were determined using unpaired *t*-test. For multiple comparisons, one-way or two-way analysis of variance (ANOVA) tests with Sidak’s corrections were used, as indicated in the figure legend. To avoid Log(0), i.e., when no colonies were recovered, CFU counts were assigned as 1. Where competitive index was calculated, this was done by dividing log_10_ transformed CFU values of predator bacteria by log_10_ transformed CFU values of prey bacteria. A value of 1 indicates no competitive advantage. Where conjugation efficiency was calculated, this was done by dividing log_10_ transformed CFU values of transconjugants by log_10_ transformed CFU values of recipient bacterial population and multiplying by 100.

### Reporting summary

Further information on research design is available in the Nature Portfolio Reporting Summary linked to this article.

## Supplementary information

Supplementary Information

Description of Additional Supplementary Files

Supplementary Software

Supplementary Movie 1

Supplementary Movie 2

Supplementary Movie 3

Supplementary Movie 4

Supplementary Movie 5

Supplementary Movie 6

Supplementary Movie 7

Reporting Summary

Transparent Peer Review file

## Source data

Source Data File

## Data Availability

Source data are provided with this paper.

## Code availability

Code for the mathematical model is provided as a Supplementary Software file.
