# Supplementary material for: Interspecies quorum sensing signals modulate multicellular organization and enhance contact-dependent antagonism in Vibrio cholerae
Source: Nat Commun. 2026 Jun 8;17:7273. doi: 10.1038/s41467-026-74086-w (PMC13402604; doi:10.1038/s41467-026-74086-w)
Supplement: Supplementary file 1 — Supplementary Information [file 41467_2026_74086_MOESM1_ESM.pdf]

Supplemental Figures

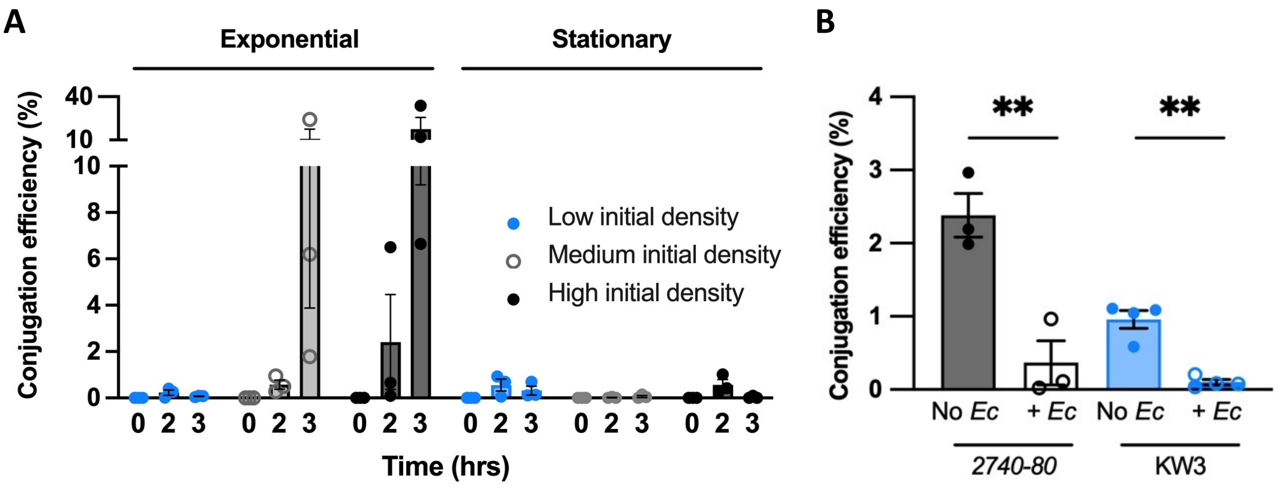

Supplementary Figure S1. Conjugation assay setup

(A) Conjugation efficiency between *V. cholerae* C6706 wild type (WT) donor (Cm^S, $J13^+$), and recipient (Cm^R) at 0-, 2- and 3- hours (hrs) post-mixing. Donor and recipient cultures were grown to exponential phase (OD_{600nm} ~0.4) or stationary phase (OD_{600nm} ~1.0) before being concentrated to OD 0.1 (low initial density) OD 1 (medium initial density) or OD 10 (high initial density).

(B) Conjugation efficiency between donor (Cm^S, $J13^+$) and recipient (Cm^R) *2740-80* or KW3 WT at 3 hours post-mixing. *E. coli* was added at a 1:1 ratio (total *V. cholerae* : *E. coli*) (+ *Ec*) or an equivalent volume of fresh LB (No *Ec*). Significance was assessed using two-sided unpaired t-test (No *Ec* vs + Ec *2740-80*, p=0.009; No *Ec* vs + Ec KW3, p=0.001).

For both panels, bars indicate mean ± SEM. Source Data are provided as a Source Data File.

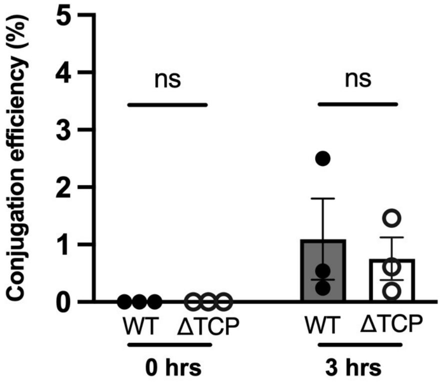

Supplementary Figure S2. ***V. cholerae*** **conjugation is unaffected by TCP aggregation when plated on solid media**
Conjugation efficiency between wild type *V. cholerae* C6706 donor (Cm^S, $J13^+$) and recipient (Cm^R) (WT) or ΔTCP *V. cholerae* donor (Cm^S, $J13^+$) and recipient (Cm^R) at 0- or 3- hrs post-mixing and plating on a solid agar pad. Donor and recipient were mixed at a 1:1 ratio before spotting on a solid agar pad. Significance was assessed using two-sided unpaired t-test. Bars indicate mean ± SEM, ns (non-significant). Source Data are provided as a Source Data File.

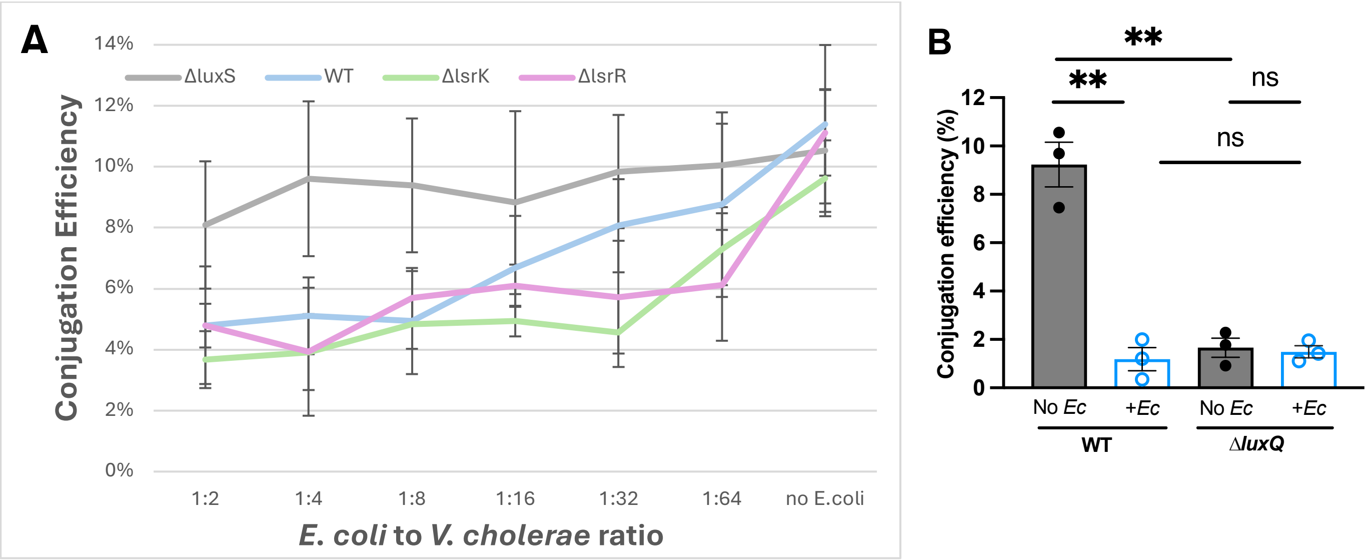

Supplementary Figure S3. ***V. cholerae*** **aggregation requires** ***luxQ*** **to be sensitive to the level of AI-2 production**
(A) Conjugation efficiency between *V. cholerae* wild type donor (Cm^S, $J13^+$), and recipient (Cm^R) at 3 hours post-mixing. *E. coli* was titrated in at ratios of 1:2, 1:4, 1:8, 1:16, 1:32, 1:64 (*E. coli* to total *V. cholerae*). An equal volume of LB was added in the instance where no *E. coli* was added (No *E.coli*). n=6 biological replicates were performed for ΔluxS, WT, and ΔlsrR. n=5 ΔlsrK. Bars indicate mean ± Stdev.
(B) Conjugation efficiency between wild type *V. cholerae* donor (Cm^S, $J13^+$) and recipient (Cm^R) (WT) or Δ*luxQ* *V. cholerae* donor (Cm^S, $J13^+$) and recipient (Cm^R) at 3 hrs post-mixing in liquid media. Significance was assessed using two-sided unpaired t-test (No *Ec* vs + Ec WT, p=0.0046; WT vs Δ*luxQ*, p=0.0068). Bars indicate mean ± SEM, ns (non-significant). Source Data are provided as a Source Data File.

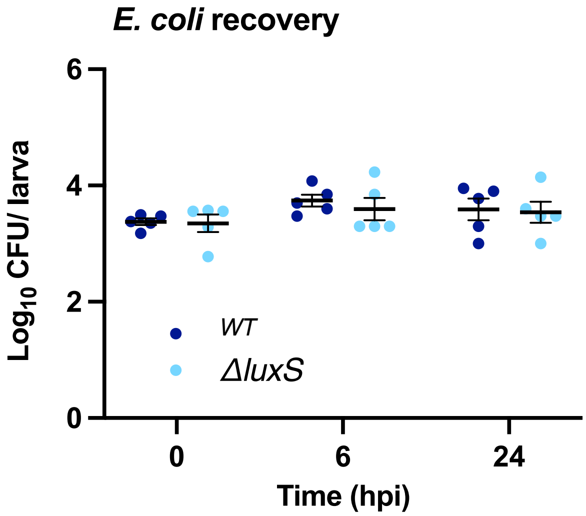

Supplementary Figure S4. *luxS* does not affect *E. coli* colonization of zebrafish HBV

Enumeration of recovered *E. coli* at 0-, 6-, or 24- hours post infection (hpi) from larvae mono-infected with either wild type (WT) or mutant (ΔluxS) *E. coli.* Data shown are from 5 larvae per time point for both WT and the ΔluxS mutant. Bars indicate mean ± SEM. Source Data are provided as a Source Data File.

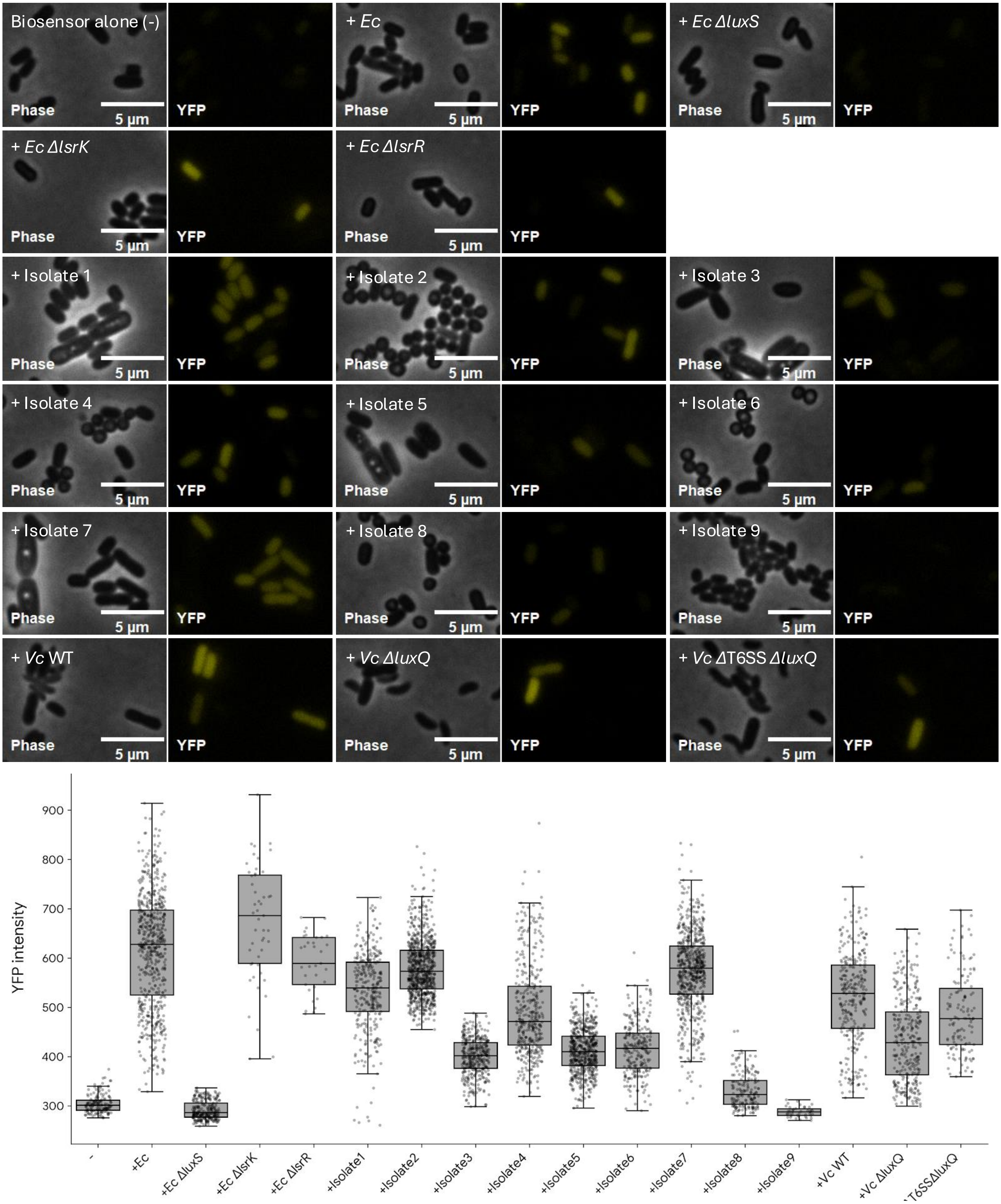

Supplementary Figure S5. Quantification of AI-2 production of strains used in this study

AI-2 production was quantified by measured using an AI-2 biosensor strain expressing YFP in the presence of AI-2. Fluorescence microscopy was used to detect and quantify YFP expression. Mean fluorescence of individual biosensor cells grown in co-culture with each tested bacterial strain. *V. cholerae* strain used is C6706. The boxes indicate the median and upper/lower quartile with wiskers at 1.5 the interquartile range. Source Data are provided as a Source Data File.

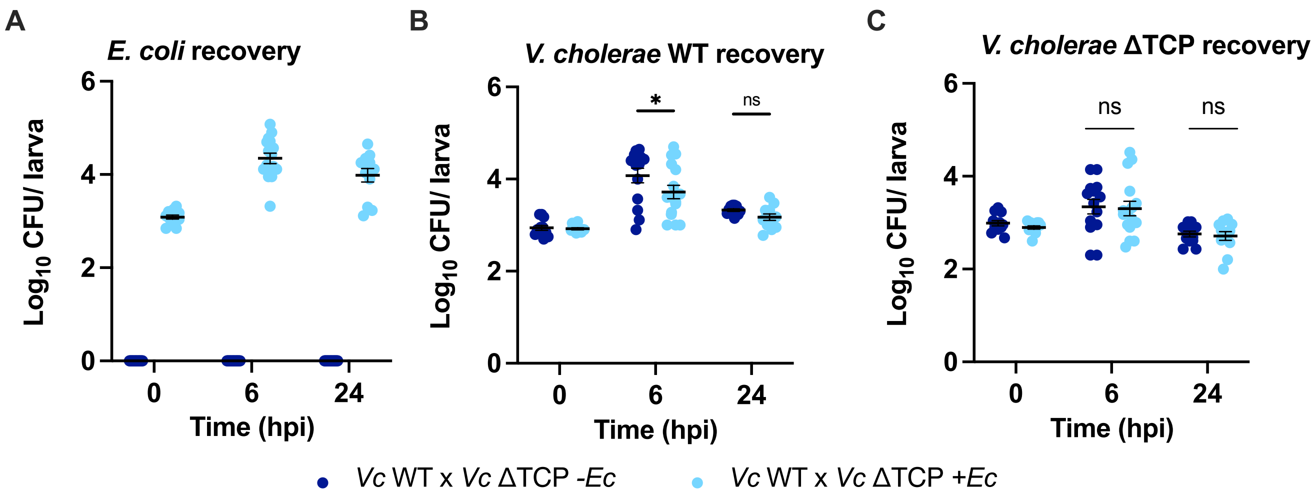

Supplementary Figure S6. *V. cholerae* aggregation contributes to colonization of the zebrafish HBV

Enumeration of recovered *E. coli* **(A)**, *V. cholerae* wild type **(B)**, or *V. cholerae* ΔTCP **(C)** at 0-, 6-, or 24- hours post infection (hpi) from larvae co-infected with a 1:1 mixture of *V. cholerae* WT and ΔTCP (*Vc* WT x *Vc* ΔTCP -*Ec*) (~750 CFU each) or a 1:1:2 mixture of *V. cholerae* WT and ΔTCP (~750 CFU each) and *E. coli* (~1500 CFU) (*Vc* WT x *Vc* ΔTCP +*Ec*). *V. cholerae* strain used is C6706. Data were pooled from four independent experiments, each with 3-4 larvae per time point (total n=12-14 for each time point). Bars indicate mean ± SEM. Statistical significance was assessed using two-way ANOVA (-*Ec* vs +*Ec* WT at 6 hpi, p=0.04). Source Data are provided as a Source Data File.

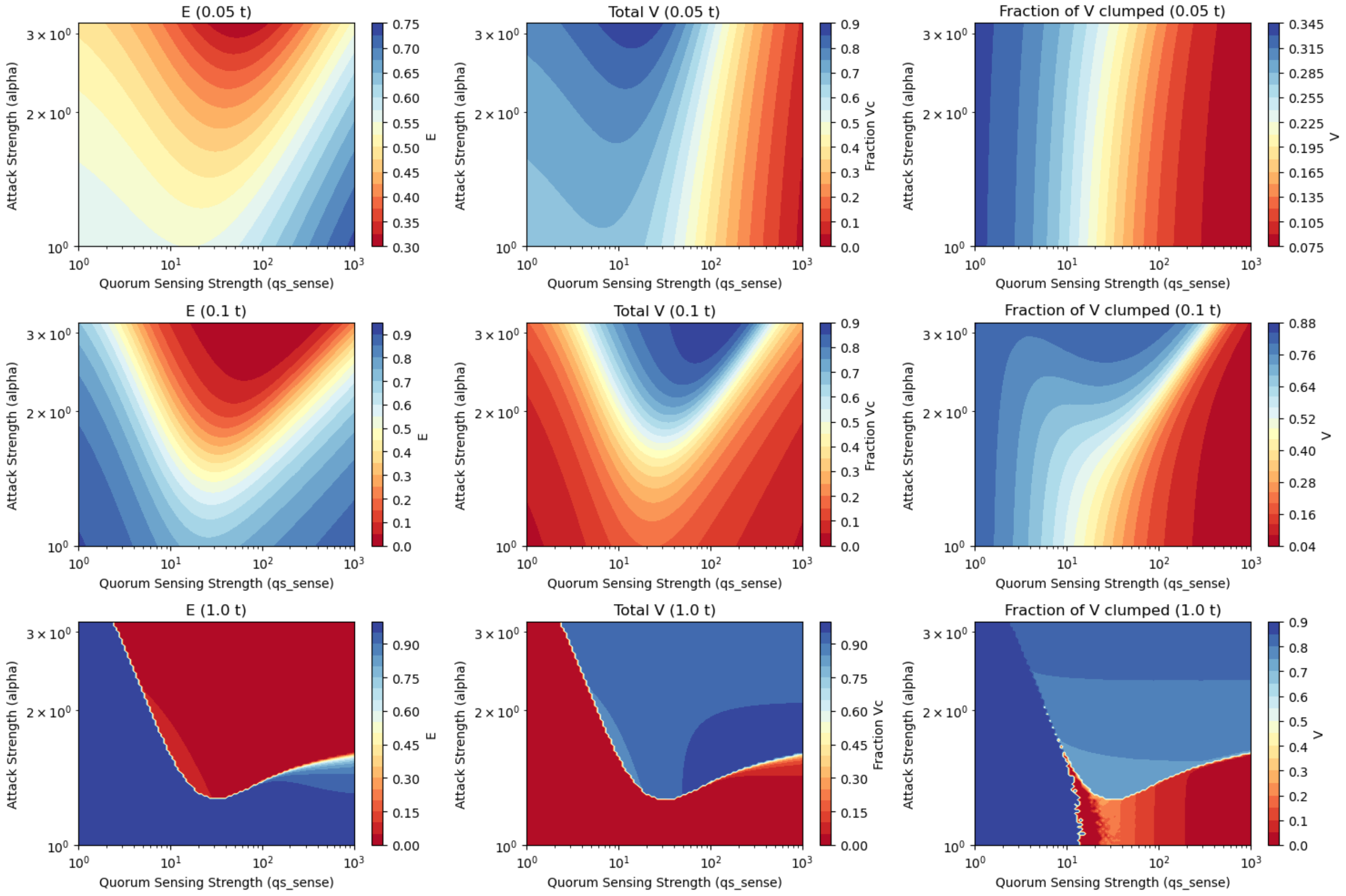

Supplementary Figure S7. Simulations sampled at intervals prior to steady state shown in Figure 7

Each row represents a time interval, increasing downwards. Left column: Total *E. coli*, Middle: Total *V. cholerae*, Right: Fraction of *V. cholerae* cells in aggregated form. Axes are explained in the text and Figure 7. Parameters are the same as the steady state shown in Figure 7, and outlined in Supplementary File S1. In this example, the fraction of *V. cholerae* cells that are in the aggregated form is set quickly by the sensitivity of the quorum sensing strength (top right). Once steady state is reached, in scenarios where *V. cholerae* dominates*,* most *V. cholerae* cells are in the aggregated form. *Note*: Where *V. cholerae* cell numbers are close to zero, the fraction of *V. cholerae* cells in the aggregated form is not meaningful.

Supplementary Table S1: Bacterial stains

ORGANISM	REF IN TEXT	RELEVANT GENOTYPE	PLASMID	RELEVANT FEATURES	SOURCE
V. CHOLERAE **C6706**	-	Str^R		Parental strain.	[1]
	Vc WT	Str^R, *vc0729::TnFGL3* (Kan^R)		Kan^R inserted into *vc0729* via transposon. *vc0729* is a neutral gene [2,3]	[1]
	Vc WT Recipient	Str^R, Cm^R, *vc0729::TnFGL3* (Kan^R)	pBAD33	*Vc* C6706 WT with Cm marker.	This Study
	Vc WT J13	Str^R, Cb^R, vc0729::TnFGL3 (Kan^R)	J13	*Vc* C6706 WT carrying the conjugative P plasmid (J13).	This Study; J13 from [4]
	Vc ΔluxQ	Str^R, *vc0729::TnFGL3* (Kan^R) Δ*luxQ*		luxQ (*vca0736*) was deleted from Vc WT carrying Tn insertion into *vc0729*	This Study
	Vc ΔluxQ Recipient	Str^R, Cm^R, *vc0729::TnFGL3* (Kan^R) Δ*luxQ*	pBAD33	*Vc* ΔluxQ with Cm marker.	This Study
	Vc luxQ J13	Str^R, Cb^R, vc0729::TnFGL3 (Kan^R) Δ*luxQ*	J13	*Vc* ΔluxQ carrying the conjugative P plasmid (J13).	This Study
	*Vc*Red	Str^R, Cb^R, *vc0729::TnFGL3* (Kan^R)	pTAC-sfCherry	*Vc* WT expressing sfCherry	This Study
	*Vc*Green	Str^R, Cb^R, *vc0729::TnFGL3* (Kan^R)	pTAC-mNeonGreen	*Vc* WT expressing mNeonGreen	This Study
	*Vc*ΔT6SS	Str^R, *vipA::TnFGL3* (Kan^R)		Kan^R inserted into *vipA* (vca0107) via transposon.	[1]
	*Vc*ΔT6SS Recipient	Str^R, Cm^R, vipA::TnFGL3 (Kan^R)	pBAD33	*Vc* ΔT6SS carrying the pBAD33.	This Study
	*Vc*ΔT6SS J13	Str^R, Cb^R, *vipA::TnFGL3* (Kan^R)	J13	*Vc* ΔT6SS carrying the conjugative P plasmid (J13).	This Study
	*Vc*ΔTCP	Str^R, *tcpC::TnFGL3* (KanR)		Kan^R inserted into *tcpC* (vc0831) via transposon.	[1]
	*Vc*ΔTCP Recipient	Str^R, Cm^R, *tcpC::TnFGL3* (KanR)	pBAD33	*Vc* ΔTCP with Cm marker	This Study
	*Vc*ΔTCP J13	Str^R, Cb^R, *tcpC::TnFGL3* (Kan^R)	J13	*Vc* ΔTCP carrying the conjugative P plasmid (J13).	This Study
	*Vc*ΔTCP Red	Str^R, Cb^R, *tcpC::TnFGL3* (KanR)	pTAC-sfCherry	*Vc* ΔTCP carrying the pBAD24-OriT-sfCherry fluorescent plasmid.	This Study
	*Vc*ΔTCP Green	Str^R, Cb^R, *tcpC::TnFGL3* (KanR)	pTAC-mNeonGreen	*Vc* ΔTCP carrying the pBAD24 - mNeonGreen fluorescent plasmid.	This Study
	Vc mshA::TnFLG3	Str^R, *mshA::TnFGL3* (Kan^R)		Kan^R inserted into *mshA* (vc0409) via transposon.	[1]
	Vc mshA::TnFLG3 Recipient	Str^R, Cm^R, *mshA::TnFGL3* (KanR)	pBAD33	*Vc mshA*::TnFLG3 with Cm marker	This Study
	Vc mshA::TnFLG3 J13	Str^R, Cb^R, *mshA::TnFGL3* (Kan^R)	J13	*Vc mshA*::TnFLG3 carrying the conjugative P plasmid (J13).	This Study
	Vc pilA::TnFLG3	Str^R, *pilA::TnFGL3* (Kan^R)		Kan^R inserted into *pilA* (vc2423) via transposon.	[1]
	Vc pilA::TnFLG3 Recipient	Str^R, Cm^R, *pilA::TnFGL3* (Kan^R)	pBAD33	*Vc pilA*::TnFLG3 with Cm marker	This Study
	Vc pilA::TnFLG3 J13	Str^R, Cb^R, *pilA::TnFGL3* (Kan^R)	J13	*Vc pilA*::TnFLG3 carrying the conjugative P plasmid (J13).	This Study
	Vc flaA::TnFLG3	Str^R, *flaA::TnFGL3* (Kan^R)		Kan^R inserted into *flaA* (vc2188) via transposon.	[1]
	Vc flaA::TnFLG3 Recipient	Str^R, Cm^R, *flaA::TnFGL3* (Kan^R)	pBAD33	*Vc flaA*::TnFLG3 with Cm marker	This Study
	Vc flaA::TnFLG3 J13	Str^R, C^R, *flaA::TnFGL3* (Kan^R)	J13	*Vc flaA*::TnFLG3 carrying the conjugative P plasmid (J13).	This Study
	Vc waaL::TnFLG3	Str^R, *waaL::TnFGL3* (Kan^R)		Kan^R inserted into *waaL* (vc0237) via transposon.	[1]
	Vc waaL::TnFLG3 Recipient	Str^R, Cm^R, waaL::TnFGL3 (Kan^R)	pBAD33	*Vc waaL*::TnFLG3 with Cm marker	This Study
	Vc waaL::TnFLG3 J13	Str^R, Cb^R, *waaL::TnFGL3* (Kan^R)	J13	*Vc* C6706 *waaL*::TnFLG3 carrying the conjugative P plasmid (J13).	This Study
	Vc vpsL::TnFLG3	Str^R, *vpsL::TnFGL3* (Kan^R)		Kan^R inserted into *vpsL* (vc0934) via transposon.	[1]
	Vc vpsL::TnFLG3 Recipient	Str^R, Cm^R, *vpsL::TnFGL3* (Kan^R)	pBAD33	*Vc* C6706 *vpsL*::TnFLG3 with Cm marker	This Study
	Vc vpsL::TnFLG3 J13	Str^R, Cb^R, *vpsL::TnFGL3*, (Kan^R)	J13	*Vc* C6706 *vpsL*::TnFLG3 carrying the conjugative P plasmid (J13).	This Study
	Vc vpsA::TnFLG3	Str^R, *vpsA::TnFGL3*, (Kan^R)		Kan^R inserted into *vpsA* (vc0917) via transposon.	[1]
	Vc vpsA::TnFLG3 Recipient	Str^R, Cm^R, *vpsA::TnFGL3* (Kan^R)	pBAD33	*Vc* C6706 *vpsA*::TnFLG3 with Cm marker	This Study
	Vc vpsA::TnFLG3 J13	Str^R, Cb^R, *vpsA::TnFGL3,* (Kan^R)	J13	*Vc* C6706 *vpsA*::TnFLG3 carrying the conjugative P plasmid (J13).	This Study
	*Vc*ΔTCPΔvpsLΔpilA	Str^R, *tcpC::TnFGL3* (KanR) ΔvpsLΔpilA		Kan^R inserted into *tcpC* via transposon. Clean deletion of *vpsL* and *pilA*	This Study

	*Vc*ΔTCPΔvpsLΔpilA Recipient	Str^R, Cm^R, *tcpC::TnFGL3* (KanR) ΔvpsLΔpilA	pBAD33	*Vc* C6706 ΔTCPΔvpsLΔpilA with Cm marker	This Study
	*Vc*ΔTCPΔvpsLΔpilA J13	Str^R, Cb^R, *tcpC::TnFGL3* (KanR) ΔvpsLΔpilA	J13	*Vc* C6706 ΔTCPΔvpsLΔpilA carrying the conjugative P plasmid (J13).	This Study
V. CHOLERAE 2740-80	-	Str^R		Parental strain.	[5]
	-	Str^R, Δ*vipA*		T6SS mutant of parental strain.	[5]
	-	Str^R, *vc1520::TnFGL3*, (Kan^R)		Kan^R inserted into *vc1520* via transposon. *vc1520* is a neutral gene [2,3]	[6]
	2740-80 WT	Str^R, *vc1520::TnFGL3,* (Kan^R), Cb^R	pBAD24	WT with Carb marker	This Study
	-	Str^R, *vc1520::TnFGL3* (Kan^R) Δ*vipA*		Kan^R inserted into *vc1520* via transposon.	[6]
	2740-80 ΔT6SS	Str^R, Cb^R, *vc1520::TnFGL3* (Kan^R) Δ*vipA,*	pBAD24	ΔT6SS with Carb marker	This Study
	2740-80 J13	Str^R, Cb^R, *vc1520::TnFGL3* (Kan^R)	J13	WT carrying the conjugative P plasmid (J13).	This study
	2740-80 Recipient	Str^R, Cm^R, *vc1520::TnFGL3* (Kan^R)	pBAD33	WT with Cm marker.	This study
	2740-80 Red	Str^R, Cb^R, *vc1520::TnFGL3,* (Kan^R)	pTAC-sfCherry	WT expressing sfCherry.	This study
***V. CHOLERAE* KW3**	-	Str^R		Parental strain.	[7]
	KW3 J13	Str^R, Cb^R	J13	*Vc* Haiti WT carrying the conjugative P plasmid (J13).	This study
	KW3 Recipient	Str^R, Cm^R,	pBAD33	*Vc* Haiti WT with Cm marker	This study
***E. COLI* DH10B**	*Ec*	Str^R, Cm^R	pTAC-mNeonGreen	Constitutive mNeonGreen expression	[6]
	Ec Δ*luxS*	*luxS*::Kan^R		Kan disruption of *luxS* via *λ*red recombination.	This study
	EcΔlsrK	lsrK::Cm^R		CmR disruption of *lsrK* via *λ*red recombination.	This study
	EcΔlsrR	lsrR::Cm^R		CmR disruption of *lsrR* via *λ*red recombination.	This study
	*Ec*Green Δ*luxS*	luxS::Kan^R, Cm^R	pTAC-mNeonGreen	Δ*luxS* mutant with Constitutive mNeonGreen expression	This study
**VARIOUS SPECIES ISOLATED FROM MOUSE FECES OR INTESTINAL SAMPLES. SEE FIG. 5 FOR DETAILS**	Mouse isolate 1				This study
	Mouse isolate 2				This study
	Mouse isolate 3				This study
	Mouse isolate 4				This study
	Mouse isolate 5				This study
	Mouse isolate 6				This study
	Mouse isolate 7				This study
	Mouse isolate 8				This study
	Mouse isolate 9				This study

Supplementary Table S2: Primers used

Primer	Sequence	Purpose	Source
**luxS_F**	GAGGTGGCTAAATGCCGTTGTTAGATAGCTTCACAGTCGATCATACC CGGATGGAAGCGCGTGTAGGCTGGAGCTGCTTC	Primers to amplify the amplify the kanamycin resistance cassette from pKD4[8] with homology regions to *luxS*.	This study
**luxS_R**	GATAGTTTACTGACTAGATGTGCAGTTCCTGCAACTTCTCTTTCGGC AGTGCCAGTTCTTATATGAATATCCTCCTTAG		
**luxSconf_F**	GCGCGTCTTTCATATACTCAGA	Primers to confirm disruption of *luxS*.	This study
**luxSconf_R**	GGTGCGCACTAAGTACAACTA		
**lsrK_F**	GGCTCGACTCTTTACCCTTTCAGAATCAAAGTACTACCTGATGGCGC TGGATGCAGGCACCGGAAGTAAACCCATCACATATACCTGCCG	Primers to amplify the amplify the chloramphenicol resistance cassette from pDS132[9] with homology regions to the *lsrK*.	This study
**lsrK_R**	GGTCATCATGATGTGGCTGTCAATGAAACTATAACCCAGGCGCTTTC CATAACGACGAGCCATGACCCGGGAATTAC		
**lsrKconf_F**	GGACGTTCCTGGTTCTATGGAA	Primers to confirm disruption of *luxS*.	This study
**lsrKconf_R**	ACAGCCACGGTCATCATGA		
**lsrR_F**	AATGAATTATGACAATCAACGATTCGGCAATTTCAGAACAGGGAAT GTGTGAAGAAGAACAGGTCGCGAACCCATCACATATACCTGCCG	Primers to amplify the amplify the chloramphenicol resistance cassette from pDS132[9] with homology regions to the *lsrR*.	This study
**lsrR_R**	CTCCATCATTCCCGGTAATAAGGTCATGCAAATTTAACTACGTAAAAT CGCCGCTGCTGTGTCAGCCATGACCCGGGAATTAC		
**lsrRconf_F**	CGGCAATTTCAGAACAGGGAATG	Primers to confirm disruption of *luxS*.	This study
**lsrRconf_R**	TCGGCTTATGCGTTCTGTCT		
**V1V2_F**	AGAGTTTGATCATGGCTCAG	Primers to amplify 16s variable region.	[10]
**V6V9_R**	TACGGCTACCTTGTTACGACTT		
***tcpP*_F**	ATGGGGTATGTCCGCGTGAT	qRT-PCR primers for *tcpP*	This study
***tcpP*_R**	TGATCATTTGGACAGGGGGC		
***dnaB*_F**	GCAGCTATGGACTGACGGTT	qRT-PCR primers for *dnaB*	[11]
***dnaB*_R**	ATGCCTCTTCCAATACCCGC		
***toxT*_F**	GCGTTGGGCAGATATTTGTGG	qRT-PCR primers for *toxT*	This study
***toxT*_R**	CGCTAGCAAACCCAGACTGA		
***toxR*_F**	GATTAGGCAGCAACGAAAGC	qRT-PCR primers for *toxR*	[12]
***toxR*_R**	AATCACCTCGTTTGGACGTT		
***tcpH*_F**	GCTTACCCAGACCGATCCAC	qRT-PCR primers for *tcpH*	This study
***tcpH*_R**	GGGAAGGCGAGAAAACAACC		
***tcpA*_F**	TGGGGATTTTCTCATTTCCA	qRT-PCR primers for *tcpA*	[13]
***tcpA*_R**	CTTCCTGGTGCAATGGACTT		
***tcpC*_F**	CTCCGCCTCAGCAAGATTAG	qRT-PCR primers for *tcpC*	[13]
***tcpC*_R**	TCGGCATAACCATCATGCTA		
***tcpD*_F**	CTGGGAGGTGGAGCCTATTT	qRT-PCR primers for *tcpD*	[13]
***tcpD*_R**	AAAAACCAGTTAGCGCTTGGT		
***hapA*_F**	TGCTCAGTAAAACCGTGGCT	qRT-PCR primers for *hapA*	This study
***hapA*_R**	TGGTTGAATACTTCGCCGCT		
***hapR*_F**	AGGCTGGCCATGTTATCGAC	qRT-PCR primers for *hapR*	This study
***hapR*_R**	GACCGATTGTCACTGGCTCA		

Supplementary References

1 Cameron, D. E., Urbach, J. M. & Mekalanos, J. J. A defined transposon mutant library and its use in identifying motility genes in Vibrio cholerae. *Proc Natl Acad Sci U S A* **105**, 8736-8741 (2008). https://doi.org/10.1073/pnas.0803281105

2 Pritchard, J. R. *et al.* ARTIST: high-resolution genome-wide assessment of fitness using transposon-insertion sequencing. *PLoS Genet* **10**, e1004782 (2014). https://doi.org/10.1371/journal.pgen.1004782

3 Fu, Y., Waldor, M. K. & Mekalanos, J. J. Tn-Seq analysis of Vibrio cholerae intestinal colonization reveals a role for T6SS-mediated antibacterial activity in the host. *Cell Host Microbe* **14**, 652-663 (2013). https://doi.org/10.1016/j.chom.2013.11.001

4 Fu, Y., Ho, B. T. & Mekalanos, J. J. Tracking Vibrio cholerae Cell-Cell Interactions during Infection Reveals Bacterial Population Dynamics within Intestinal Microenvironments. *Cell Host Microbe* **23**, 274-281 e272 (2018). https://doi.org/10.1016/j.chom.2017.12.006

5 Basler, M., Pilhofer, M., Henderson, G. P., Jensen, G. J. & Mekalanos, J. J. Type VI secretion requires a dynamic contractile phage tail-like structure. *Nature* **483**, 182-186 (2012). https://doi.org/10.1038/nature10846

6 Virgo, M., Mostowy, S. & Ho, B. T. Use of zebrafish to identify host responses specific to type VI secretion system mediated interbacterial antagonism. *PLoS Pathog* **20**, e1012384 (2024). https://doi.org/10.1371/journal.ppat.1012384

7 Bashir, A. *et al.* A hybrid approach for the automated finishing of bacterial genomes. *Nat Biotechnol* **30**, 701-707 (2012). https://doi.org/10.1038/nbt.2288

8 Datsenko, K. A. & Wanner, B. L. One-step inactivation of chromosomal genes in Escherichia coli K-12 using PCR products. *Proc Natl Acad Sci U S A* **97**, 6640-6645 (2000). https://doi.org/10.1073/pnas.120163297

9 Philippe, N., Alcaraz, J. P., Coursange, E., Geiselmann, J. & Schneider, D. Improvement of pCVD442, a suicide plasmid for gene allele exchange in bacteria. *Plasmid* **51**, 246-255 (2004). https://doi.org/10.1016/j.plasmid.2004.02.003

10 Johnson, J. S. *et al.* Evaluation of 16S rRNA gene sequencing for species and strain-level microbiome analysis. *Nat Commun* **10**, 5029 (2019). https://doi.org/10.1038/s41467-019-13036-1

11 Menendez-Gil, P. *et al.* Modulation of Vibrio cholerae gene expression through conjugative delivery of engineered regulatory small RNAs. *J Bacteriol* **206**, e0014224 (2024). https://doi.org/10.1128/jb.00142-24

12 Acosta, N., Pukatzki, S. & Raivio, T. L. The Cpx system regulates virulence gene expression in Vibrio cholerae. *Infect Immun* **83**, 2396-2408 (2015). https://doi.org/10.1128/IAI.03056-14

13 Yan, J. *et al.* The Response Regulator VC1795 of Vibrio Pathogenicity Island-2 Contributes to Intestinal Colonization by Vibrio cholerae. *Int J Mol Sci* **24** (2023). https://doi.org/10.3390/ijms241713523
