# Supplementary material for: Interspecies quorum sensing signals modulate multicellular organization and enhance contact-dependent antagonism in Vibrio cholerae
Source: Nat Commun. 2026 Jun 8;17:7273. doi: 10.1038/s41467-026-74086-w (PMC13402604; doi:10.1038/s41467-026-74086-w)
Supplement: Supplementary file 2 — Description of Additional Supplementary Files [file 41467_2026_74086_MOESM2_ESM.pdf]

Supplementary Movie 1. WT larvae infected in the HBV with *Vc*Red and *Vc*Green, 6 hours post infection
Wild type AB larvae were injected in the hindbrain ventricle (HBV) with ~750 CFU wild type *V. cholerae* C6706 constitutively expressing *sfCherry* (*Vc*Red) and ~750 CFU wild type *V. cholerae* C6706 constitutively expressing *mNeonGreen* (*Vc*Green). Larvae were imaged at 40x magnification. Representative Z-stack from single larvae at 6 hours post infection shown. Scale bar, 10 µm.

Supplementary Movie 2. WT larvae infected in the HBV with *Vc*Red, *Vc*Green and *E. coli,* 6 hours post infection
Wild type AB larvae were injected in the hindbrain ventricle (HBV) with ~750 CFU wild type *V. cholerae* C6706 constitutively expressing *sfCherry* (*Vc*Red) and ~750 CFU wild type *V. cholerae* C6706 constitutively expressing *mNeonGreen* (*Vc*Green) and ~1500 CFU *E. coli* (no color). Larvae were imaged at 40x magnification. Representative Z-stack from single larvae at 6 hours post infection shown. Scale bar, 10 µm.

Supplementary Movie 3. WT larvae infected in the HBV with *Vc*ΔTCP Red and *Vc*ΔTCP Green, 6 hours post infection
Wild type AB larvae were injected in the hindbrain ventricle (HBV) with ~750 CFU *V. cholerae* C6706ΔTCP constitutively expressing *sfCherry* (*Vc*ΔTCP Red) and ~750 CFU *V. cholerae* C6706ΔTCP constitutively expressing *mNeonGreen* (*Vc*ΔTCP Green). Larvae were imaged at 40x magnification. Representative Z-stack from single larvae at 6 hours post infection shown. Scale bar, 10 µm.

Supplementary Movie 4. WT larvae infected in the HBV with *Vc*ΔTCP Red and *Vc*Green, 6 hours post infection
Wild type AB larvae were injected in the hindbrain ventricle (HBV) with ~750 CFU *V. cholerae* C6706ΔTCP constitutively expressing *sfCherry* (ΔTCP Red) and ~750 CFU wild type *V. cholerae* C6706 constitutively expressing *mNeonGreen* (*Vc*Green). Larvae were imaged at 40x magnification. Representative Z-stack from single larvae at 6 hours post infection shown. Scale bar, 10 µm.

Supplementary Movie 5. WT larvae infected in the HBV with *Vc*ΔTCP Green and *Vc*Red, 6 hours post infection
Wild type AB larvae were injected in the hindbrain ventricle (HBV) with ~750 CFU wild type *V. cholerae* C6706 constitutively expressing *sfCherry* (*Vc*Red) and ~750 CFU *V. cholerae* C6706ΔTCP constitutively expressing *mNeonGreen* (*Vc*ΔTCP Green). Larvae were imaged at 40x magnification. Representative Z-stack from single larvae at 6 hours post infection shown. Scale bar, 10 µm.

Supplementary Movie 6. WT larvae infected in the HBV with *2740-80*Red and *Ec*Green Δ*luxS*, 6 hours post infection
Wild type AB larvae were injected in the hindbrain ventricle (HBV) with ~1500 CFU wild type *V. cholerae 2740-80* constitutively expressing *sfCherry* (*Vc*Red) and 1500 *E. coli* Δ*luxS* constitutively *mNeonGreen* (*Ec*Green Δ*luxS*). Larvae were imaged at 40x magnification. Representative Z-stack from single larvae at 6 hours post infection shown. Scale bar, 10 µm.

Supplementary Movie 7. WT larvae infected in the HBV with *2740-80*Red and *Ec*Green, 6 hours post infection
Wild type AB larvae were injected in the hindbrain ventricle (HBV) with ~1500 CFU wild type *V. cholerae 2740-80* constitutively expressing *sfCherry* (*Vc*Red) and 1500 *E. coli* wild type constitutively *mNeonGreen* (*Ec*Green WT). Larvae were imaged at 40x magnification. Representative Z-stack from single larvae at 6 hours post infection shown. Scale bar, 10 µm.

Supplementary Software. Detailed description of computational model for aggregation and T6SS dynamics

Supplementary Information.
Contains Supplementary Figures S1-S7, Supplementary Table S1,S2
