# Supplementary material for: Interspecies quorum sensing signals modulate multicellular organization and enhance contact-dependent antagonism in Vibrio cholerae
Source: Nat Commun. 2026 Jun 8;17:7273. doi: 10.1038/s41467-026-74086-w (PMC13402604; doi:10.1038/s41467-026-74086-w)
Supplement: Supplementary file 3 — Supplementary Software [file 41467_2026_74086_MOESM3_ESM.zip › Mathematical Model.pdf]

Model Description

This model describes the population dynamics of three microbial groups:

- **E**: E. coli
- **F**: Free-living Vibrio
- **C**: Clumped Vibrio

It is a generalized competitive Lotka-Volterra system with additional transitions between F and C, modulated by quorum sensing.

State Variables

- (E): Population of E. coli
- (F): Population of free-living Vibrio
- (C): Population of clumped Vibrio

Parameters

- (r_E, r_F, r_C): Intrinsic growth rates of E, F, and C
- (K_E, K_F, K_C): Carrying capacities
- (α): Direct competition coefficient (effect of Vf on E)
- (β): symmetric competition for space (like-on-like)
- (γ): symmetric competition between clumped and free cells
- $(\mathrm{R}_{F\to C})$: Baseline rate of F to C transition
- $(\frac{\mathrm{R}_{C\to F}}{\mathrm{R}_{F\to C}})$: Ratio of baseline transition rates.
- (Q): Quorum sensing strength (how much E increases C to F transition)

Model Equations

$$\frac{dE}{dt} = r_E E \left[1 - \frac{\beta E + (\gamma + \alpha)F + \beta C}{K_E}\right]$$
$$\frac{dF}{dt} = r_F F \left[1 - \frac{\gamma E + \beta F + \gamma C}{K_F}\right] - \backslash\_\mathrm{R}_{FC} F + \mathrm{qs}\backslash\_\mathrm{R}_{CF} C$$
$$\frac{dC}{dt} = r_C C \left[1 - \frac{\beta E + \gamma F + \beta C}{K_C}\right] + \backslash\_\mathrm{R}_{FC} F - \mathrm{qs}\backslash\_\mathrm{R}_{CF} C,$$

where $\mathrm{qs}\backslash\_\mathrm{R}_{CF} = \mathrm{R}_{F\to C}\frac{\mathrm{R}_{C\to F}}{\mathrm{R}_{F\to C}} + QE$

Assumptions:

- Each population grows logistically, limited by its own and others' densities.

- Intrinsic growth rates are assumed to be the same for E and C, and lower for F, to reflect Vibrio's lower apparent rate of growth in the planktonic form.
- Carrying capacities were chosen to reflect knowledge of E. coli and Vibrio growth. The lower carrying capacity of F reflects the lower cell densities reached when Vibrio is mutated to inhibit aggregation.
- E and Vibrio (C+F) compete for resources and space. E.coli naturally forms aggregates, annd competition for space is thus assumed to be stronger between aggregated E.coli and aggregated Vibrio, thatn between Ecoli and free-Vibrio, which can penetrate into gaps between aggregates.
- In the absence of E, the majority of Vibrio is in the C form.
- Rates of transition between C and F are arbitrarily high. Ratios of rate constans representing C to F and F to C transitions are chosed to reflect observations of C and F ratios under the microscope.
- Higher E. coli density increases the rate at which clumped Vibrio revert to free-living form. The steepness of this rate dependence is given by qs_sense (referred to as Q in the manuscript)
- The efficacy of the T6SS attack is respresented as F inhibiting E but an amount alpha (referred to as alpha in the manuscript).
- All units are arbitrary.

In [6]:
```
# Harry McClelland 23-7-25; h.mcclelland@ucl.ac.uk
# Vibrio clumping model with quorum sensing


# -------- Package imports --------
import numpy as np
from scipy.integrate import solve_ivp
import matplotlib.pyplot as plt


# -------- Model parameters --------

# E and C intrinsic growth rates are the same for simplicity, with F being half that of C.:
r_E =2      # intrinsic growth rate of E (E. coli)
r_F = 0.5*r_E     # intrinsic growth rate of F (Vibrio free)
r_C = r_E     # intrinsic growth rate of C (Vibrio clumped)

# Representative carrying capacities are altered to reflect the observations:
K_E = 1.0     # carrying capacity of E
K_F = 0.5    # carrying capacity of F
K_C = 0.8    # carrying capacity of C

# interaction coefficients
log10_max_alpha = 0.5    # direct competition coefficient: effect of Vf on E (attack strength)
log10_min_alpha = 0      #  competition for space: effect of E-on-E, E-on-C, C-on-C, C-on-E, and F-on-F (like-on-like)
beta = 1.0   #
gamma = 0.5   # competition coefficient: effect of C-on-F, F-on-C, F-on-C and C-on-F (clumped vs free)

# Quorum sensing parameters
rate_F_to_C = 10.0          #  rate of Vf -> Vc transition (constant)
rate_C_to_F_ratio = 0.1     #  baseline rate of Vc -> Vf transition
log10_max_qssense = 3.0           #  quorum sensing strength (how much E increases transition rate)
log10_min_qssense = 0.0

# Simulation time
t_span = (0, 100)
t_eval = np.linspace(*t_span, 100)
```

```python
resolution = 20

# -------- Lotka-Volterra model with quorum sensing --------
# This function defines the system of ODEs for the Lotka-Volterra model with quorum sensing dynamics.
# It takes time t, state vector y, competition coefficient alpha, and quorum sensing strength qs_sense as inputs.
# The state vector y contains populations of E, Vf, and Vc.
# The function returns the derivatives of these populations with respect to time.

def lotka_volterra_qs(t, y, alpha, qs_sense):

    # Unpack the state vector
    E, F, C = y

    # Quorum sensing: Vf -> Vc transition rate constant, Vc -> Vf transition rate increases with E
    qs_rateFC = r_F*rate_F_to_C
    qs_rateCF = r_F*(rate_C_to_F_ratio * rate_F_to_C  +  qs_sense * E)

    # ODEs:
    dE_dt   =    r_E * E *  (1 - (beta* E    + (gamma + alpha)*F        + beta*C    ) / K_E)
    dF_dt   =    r_F * F *  (1 - (gamma*E    + beta *F                  + gamma*C   ) / K_F) - qs_rateFC * F + qs_rateCF * C
    dC_dt   =    r_C * C *  (1 - (beta* E    + gamma*F                  + beta*C    ) / K_C) + qs_rateFC * F - qs_rateCF * C

    return [dE_dt, dF_dt, dC_dt]

# Initial conditions
E0 = 0.002
F0 = 0.001
C0 = 0.001
y0 = [E0, F0, C0]




# Stability space: Vary alpha (attack strength) and qs_sense (quorum sensing)
alphas = np.logspace(log10_min_alpha, log10_max_alpha, resolution)
qs_senses = np.logspace(log10_min_qssense, log10_max_qssense, resolution)
E_eq = np.zeros((len(alphas), len(qs_senses)))
F_eq = np.zeros_like(E_eq)
C_eq = np.zeros_like(E_eq)

for i, a in enumerate(alphas):
    for j, qs in enumerate(qs_senses):
        sol = solve_ivp(lotka_volterra_qs, t_span, y0, args=(a, qs), t_eval=[t_span[1]])
        E_eq[i, j] = sol.y[0, -1]
        F_eq[i, j] = sol.y[1, -1]
        C_eq[i, j] = sol.y[2, -1]
        progress = (i * len(qs_senses) + j + 1) / (len(alphas) * len(qs_senses)
        )
        bar_length = 40
        filled_length = int(bar_length * progress)
        bar = '=' * filled_length + '-' * (bar_length - filled_length)
        print(f'\rProgress: [{bar}] {progress:.1%}', end='')
```

```
Progress: [========================================] 100.0%
```

Plotting:

In [7]:
```
# Compute fraction of V in the form Vc at equilibrium
V_total_eq = F_eq + C_eq
C_frac_eq = np.divide(C_eq, V_total_eq, out=np.zeros_like(C_eq), where=V_total_eq!=0)

# Clip values to [0, 1] range
E_eq_clipped = np.clip(E_eq, 0, 1)
C_frac_eq_clipped = np.clip(C_frac_eq, 0, 1)
V_total_eq_clipped = np.clip(V_total_eq, 0, 1)

fig, axs = plt.subplots(1, 3, figsize=(16, 6))

# Stability space for E
c1 = axs[0].contourf(qs_senses, alphas, E_eq_clipped, levels=20, cmap='viridis')
fig.colorbar(c1, ax=axs[0], label='Equilibrium E')
axs[0].set_xlabel('Quorum Sensing Strength (qs_sense)')
axs[0].set_ylabel('Attack Strength (alpha)')
axs[0].set_title('Equilibrium E')
axs[0].set_xscale('log')
axs[0].set_yscale('log')

# Fraction of V in the form Vc
c2 = axs[1].contourf(qs_senses, alphas, V_total_eq_clipped, levels=20, cmap='viridis')
fig.colorbar(c2, ax=axs[1], label='V')
axs[1].set_xlabel('Quorum Sensing Strength (qs_sense)')
axs[1].set_ylabel('Attack Strength (alpha)')
axs[1].set_title('Equilibrium total V')
axs[1].set_xscale('log')
axs[1].set_yscale('log')

# Fraction of V in the form Vc
c3 = axs[2].contourf(qs_senses, alphas, C_frac_eq_clipped, levels=20, cmap='viridis')
fig.colorbar(c3, ax=axs[2], label='Fraction Vc')
axs[2].set_xlabel('Quorum Sensing Strength (qs_sense)')
axs[2].set_ylabel('Attack Strength (alpha)')
axs[2].set_title('Fraction of V in form Vc at equilibrium')
axs[2].set_xscale('log')
axs[2].set_yscale('log')



plt.tight_layout()
plt.show()
```

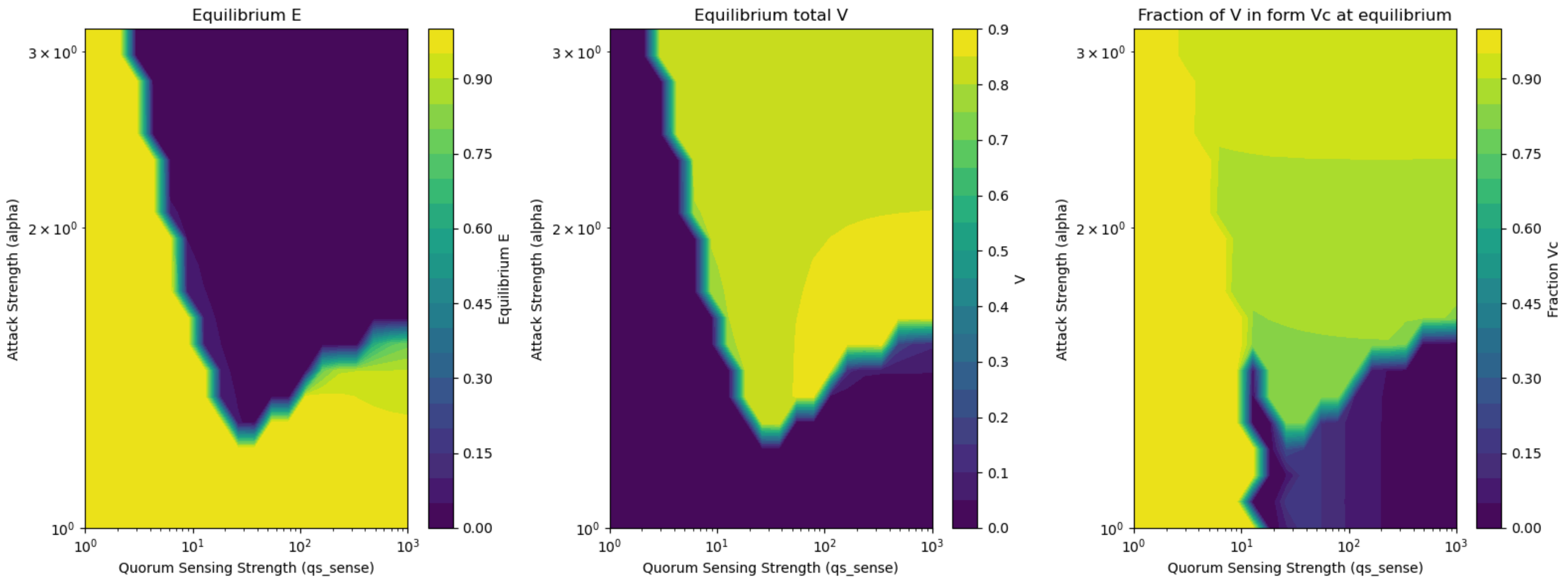

Equilibrium E
Attack Strength (alpha)
Quorum Sensing Strength (qs_sense)
Equilibrium E
0.00
0.15
0.30
0.45
0.60
0.75
0.90
Equilibrium total V
Attack Strength (alpha)
Quorum Sensing Strength (qs_sense)
V
0.0
0.1
0.2
0.3
0.4
0.5
0.6
0.7
0.8
0.9
Fraction of V in form Vc at equilibrium
Attack Strength (alpha)
Quorum Sensing Strength (qs_sense)
Fraction Vc
0.00
0.15
0.30
0.45
0.60
0.75
0.90
