# Supplementary material for: Interspecies quorum sensing signals modulate multicellular organization and enhance contact-dependent antagonism in Vibrio cholerae
Source: Nat Commun. 2026 Jun 8;17:7273. doi: 10.1038/s41467-026-74086-w (PMC13402604; doi:10.1038/s41467-026-74086-w)
Supplement: Supplementary file 12 — Transparent Peer Review file [file 41467_2026_74086_MOESM12_ESM.pdf]

nature portfolio

Peer Review File

Interspecies quorum-sensing signals modulate multicellular organization and enhance contact-dependent antagonism in Vibrio cholerae

Corresponding Author: Dr Brian Ho

This file contains all reviewer reports in order by version, followed by all author rebuttals in order by version.

Version 0:

Reviewer comments:

Reviewer #1

(Remarks to the Author)
Summary
This manuscript by Virgo et al. presents fascinating work on TCP-mediated, quorum-sensing–controlled aggregation and its role in shaping interbacterial interactions. Overall, this will be an exciting addition to the field. The paper is clearly written, straightforward to follow, and the experiments are well-executed. A few clarifications and control experiments will further strengthen the conclusions. Below I outline my specific suggestions.

Major comments:
-Line 174-175 and Fig 2A - Both vpsL and pilA mutants display mild conjugation defects. This raises the question: is aggregation and conjugation efficiency fully eliminated in the triple mutant (vpsL pilA tcp)? This could be directly relevant to the data in Fig. 4B, where aggregation in the tcp mutant is not completely abolished. If animal experiments are hard, displaying only for conjugation may still be informative.

- The data in Fig. 4D nicely demonstrate the role of T6SS. It would be helpful to also see aggregate formation for all three conditions from Fig. 4D represented in Fig. 4C. If these experiments are particularly challenging in the animal model, it would be useful to acknowledge this caveat explicitly in the text.

-In 5B – The phenotype of microbiota member driven disaggregation is clear. However, the connection to AI-2 can be made more clear. A simple validation, such as applying spent media to an AI-2 biosensor strain, would help confirm whether these bacteria indeed produce AI-2. If such tests are not feasible, the text should be revised to broaden the conclusion, acknowledging that other molecules could also explain the phenotype.

-Line 234-237 – The in vivo V. cholerae to E. coli ratio differs from the in vitro ratio (5:1). Could the luxS mutant display a phenotype in vitro if tested at the same ratios used in vivo? This is particularly relevant since higher E. coli levels would increase AI-2 availability.

- The sudden switch to mice at this stage was not well explained. Would it not have been more appropriate to first test with the natural zebrafish microbiota system? Please clarify this rationale in the text.

-Line 179-181 – In 2B (and in 1A) While large aggregates do not form, smaller aggregates are clearly present. Consider revising the text to reflect this distinction (“large/small aggregates” rather than “complete absence”). If possible, include a simple quantification of aggregate size using existing images; this should not require additional experimentation.

Minor comments:
-Line 168-171 – Please specify which mutants correspond to which processes. For example, clarify that vpsL and vpsA are linked to VPS production. This would help orient the reader.

-General comment: Different isolates of V. cholerae are used in this study. Please specify in the text which isolate was used for each experiment (e.g., was the library derived from C6706?).

-In 1A, Please clearly mark which cells are designated as assemblies versus planktonic. In the merged image, the red and green signals appear intermixed, yet the text states that they form mono communities. Please clarify.

-Strain table is nicely presented. I do recommend two additions: first, can locus tags be provided, alongside the gene names. This is helpful to community in general when following up on your work. Secondly, is the vc729 a neutral locus? Perhaps mention it somewhere in text.

-Typo – KW cholerae (the e is missing).

-Line 252-253 – Please check figure call outs. They appear to be switched.

Reviewer #2

(Remarks to the Author)
The manuscript by Virgo et al. addresses the interesting topic of the dynamic spatial organization of bacterial communities and how it affects interspecies interactions. Using Vibrio cholerae and Escherichia coli in a zebrafish colonization model, they show that the presence of E. coli affects the aggregation phenotype of Vibrio by downregulating TCP pilus expression. The authors further argue that this phenotype is AI-2-dependent. As aggregation results in less T6SS-mediated killing of competing E. coli cells, disaggregation might be a mechanism to effectively attack and eliminate the competitor. This suggests that Vibrio relies on quorum sensing to balance between colonization-promoting aggregation and active killing single-cell states.

The observations are very intriguing and would be of interest to a wide readership, and the manuscript is well written. I do, however, have several points that the authors might want to consider in order to strengthen their arguments, especially regarding the AI-2 dependency of the observed phenotype:

Figure 3. I think basing the conclusions solely on the luxS knockout may not be sufficient to make a strong point. The luxS deletion is notoriously pleiotropic and affects the expression of many genes in E. coli in an AI-2-independent manner (including motility and a large set of metabolic genes). This might ultimately also affect the colonization ability of the strain itself. It would therefore be useful to see the CFUs of E. coli WT and luxS in monocolonized zebrafish, as well as in co-colonization with Vibrio, to exclude colonization-rate–associated effects.
I would not exclude the luxS mutant from the analysis, but I would definitely add some control experiments. Utilizing more specific knockouts would help to modulate extracellular AI-2 levels. I suggest comparing the effects of E. coli lsrK (AI-2–overproducing) and/or E. coli lsrR (AI-2–consuming) strains. A luxPQ mutation in Vibrio would also be important to test in order to demonstrate that the disaggregation effect is indeed governed by AI-2.
Finally, I wonder if there is a possibility to show that it is indeed AI-2, and nothing else, that affects TCP expression in Vibrio. Adding exogenous AI-2 (commercially available) might be a way to test this.

Figure 4C. I suggest swapping the panels so that E. coli WT is shown first. Furthermore, it is somewhat difficult to see the point being conveyed (“In the absence of luxS, V. cholerae and E. coli formed distinct microcolonies of species-segregated cells (Figure 4C, Video 6). However, with WT E. coli, there were a number of dispersed individual V. cholerae cells that became intermixed among E. coli; lines 216–217”). I do agree that it looks like Vibrio forms fewer aggregates (though this somehow also seems to be the case for E. coli WT as compared to luxS), but to my eye, the populations look intermixed in both conditions. Is there a way to show it in a more pronounced way?

Figure 5. In my opinion, this argument is a bit stretched. First, it would definitely help to show the colonization levels of these species, as well as Vibrio, in your experiments. The effects might simply be due to the fact that these species successfully compete against Vibrio, preventing it from reaching the CFU/g counts that are permissive for conjugation.

As all these bacteria are known to produce AI-2, you would again need to knock out luxS in at least one of them that is genetically tractable, or instead use a species that does not produce AI-2. For example, P. aeruginosa, although not normally part of the gut microbiome, can colonize the gut under certain pathological conditions.

Figure 6. If I understand the authors correctly, Vibrio, by sensing E. coli, actively disaggregates to be able to kill competing cells more efficiently via T6SS. This poses a trade-off: aggregation is important for colonization, while disaggregation is important for eliminating competitors but at the same time decreases colonization. I wonder if the authors might test a Vibrio strain locked in TCP expression (e.g., from a constitutive promoter, so that its expression is not repressed in the presence of E. coli). If the authors’ explanation is correct, one would expect that in this scenario, Vibrio should still be less fit compared to the wild-type strain in the presence of E. coli, as the ability to dynamically switch should yield the highest fitness.

Minor points:

Figure 1: It would be interesting to see how E. coli WT (and luxS?) localizes relative to Vibrio.
Figure 2A: I suggest labeling the graph with “in vitro.” Same for Figures 4E and 4F. Small guiding labels would be helpful for the reader.
Figure 4D. I wonder how the 2740-80 T6 vs E. coli WT group looks like.

Version 1:

Reviewer comments:

Reviewer #1

(Remarks to the Author)
The authors have answered my queries and done several experiments to help address questions raised by both the referees. The updated manuscript reads nice!

I only had a minor suggestion for the authors. Rather than using the phrase knocked out, consider phrasing as deletion? Or consider adding a deletion symbol in front of the strain.

Reviewer #2

(Remarks to the Author)
I thank the authors for addressing all the comments. I am, however, not entirely convinced by the explanation provided as to why deletion of lsrK and lsrR did not lead to changes in conjugation rates. The accumulation and depletion of AI-2 in the supernatants of these mutants, respectively, represent quite substantial changes in overall AI-2 levels and can hardly be considered small. Since the main message of the manuscript is that the modulation of aggregation is (mainly) regulated by AI-2, I suggest measuring extracellular AI-2 levels in the titration experiments shown in Figure 1C to provide a clearer link between AI-2 accumulation and E. coli abundance/conjugation efficiency. I would also recommend including the data from the lsrK/lsrR mutants as supplementary material, and incorporating these results into the discussion.

Open Access This Peer Review File is licensed under a Creative Commons Attribution 4.0 International License, which permits use, sharing, adaptation, distribution and reproduction in any medium or format, as long as you give appropriate credit to the original author(s) and the source, provide a link to the Creative Commons license, and indicate if changes were made.
In cases where reviewers are anonymous, credit should be given to 'Anonymous Referee' and the source.
The images or other third party material in this Peer Review File are included in the article's Creative Commons license, unless indicated otherwise in a credit line to the material. If material is not included in the article's Creative Commons license and your intended use is not permitted by statutory regulation or exceeds the permitted use, you will need to obtain permission directly from the copyright holder.
To view a copy of this license, visit https://creativecommons.org/licenses/by/4.0/

Reviewer #1 (Remarks to the Author):

This manuscript by Virgo et al. presents fascinating work on TCP-mediated, quorum-sensing–controlled aggregation and its role in shaping interbacterial interactions. Overall, this will be an exciting addition to the field. The paper is clearly written, straightforward to follow, and the experiments are well-executed. A few clarifications and control experiments will further strengthen the conclusions. Below I outline my specific suggestions.

We thank the reviewer for their enthusiasm.

-Line 174-175 and Fig 2A - Both vpsL and pilA mutants display mild conjugation defects. This raises the question: is aggregation and conjugation efficiency fully eliminated in the triple mutant (vpsL pilA tcp)? This could be directly relevant to the data in Fig. 4B, where aggregation in the tcp mutant is not completely abolished. If animal experiments are hard, displaying only for conjugation may still be informative.

We performed the conjugation assay using the triple mutant (*vpsL pilA tcp*) as suggested by the reviewer and showed that this mutant phenotypically copies the single (*tcp*) mutant (see figure below). We added this new data to our revised Figure 2 (Lines 181-183).

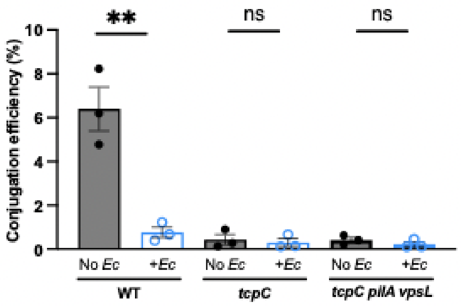

It should be noted that the *in vivo* microscopy (Figure 4B and elsewhere in our work) is valuable for observational discovery, but it is not quantitative and is therefore not the primary basis for drawing our conclusions. The apparent aggregates presented in Figure 4B that the reviewer points out may be a result of sister cells growing together in a confined space. We therefore supplemented all of our microscopy with our conjugation assays, which serves as a robust quantitative proxy measurement for aggregation.

- The data in Fig. 4D nicely demonstrate the role of T6SS. It would be helpful to also see aggregate formation for all three conditions from Fig. 4D represented in Fig. 4C. If these experiments are particularly challenging in the animal model, it would be useful to acknowledge this caveat explicitly in the text.

The other reviewer made a similar point. As suggested, we imaged bacteria mixtures of ∆T6SS *Vibrio* and *E. coli in vivo* using our zebrafish infection models. However, a significant amount of bacteria in the hindbrain was clearly localized within phagocytes/innate immune cells under these experimental conditions (see figure below):

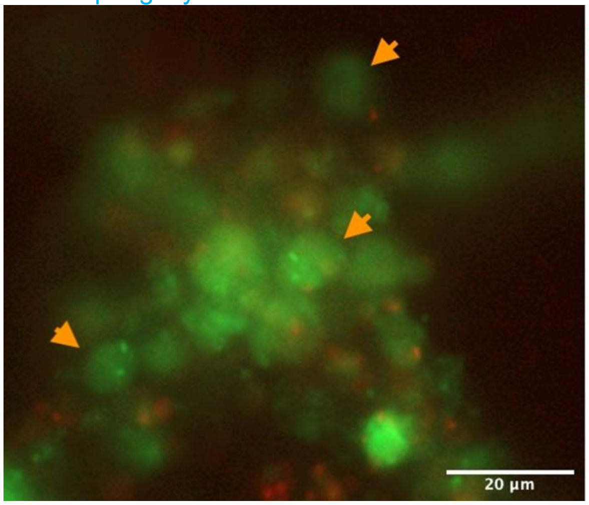

Green: *E. coli ∆luxS*

Red: *V. cholerae ∆T6*

Circular clumps of fluorescence (orange arrows) are likely to be bacteria confined within innate immune cells.

Although this result was not surprising given our previous work showing that T6SS-mediated killing by *V. cholerae* played a significant role in blocking innate immune activity (Virgo *et al*, 2024), it does make it challenging to draw inferences about aggregation for these strain combinations lacking the T6SS *in vivo*. As such, we opted to not include this microscopy in our revised manuscript and added a sentence acknowledging this limitation in the revised text (Lines 238-240).

-In 5B – The phenotype of microbiota member driven disaggregation is clear. However, the connection to AI-2 can be made more clear. A simple validation, such as applying spent media to an AI-2 biosensor strain, would help confirm whether these bacteria indeed produce AI-2. If such tests are not feasible, the text should be revised to broaden the conclusion, acknowledging that other molecules could also explain the phenotype.

We used a recently published AI-2 biosensor strain (Keizers *et al*, 2022) to test AI-2 production for each of our microbiota isolates. This new data is now presented in Supplementary Figure S5. As expected, all but one of these strains exhibited clear AI-2 production. Interestingly, a *Citrobacter* sp. isolate did not activate expression in the biosensor strain. We therefore broadened our conclusion to acknowledge the possibility that other signaling molecules may exist which also contribute to *V. cholerae* aggregation modulation (Line 265-267).

-Line 234-237 – The in vivo V. cholerae to E. coli ratio differs from the in vitro ratio (5:1). Could the luxS mutant display a phenotype in vitro if tested at the same ratios used in vivo? This is particularly relevant since higher E. coli levels would increase AI-2 availability.

We repeated these experiments with *V. cholerae* to *E. coli* ratios at 1:1. The corresponding Figures 4E/F have been updated accordingly.

- The sudden switch to mice at this stage was not well explained. Would it not have been more appropriate to first test with the natural zebrafish microbiota system? Please clarify this rationale in the text.

The key point of using mouse microbiota isolates was to demonstrate the prevalence of AI-2-producing bacteria in organisms susceptible to *V. cholerae* infection. Our zebrafish infection experiments are injections into the hindbrain, which does not carry any resident microbiota, so there are no "natural" zebrafish microbiota in our system. We revised text to make this point clear (Lines 254-256).

-Line 179-181 – In 2B (and in 1A) While large aggregates do not form, smaller aggregates are clearly present. Consider revising the text to reflect this distinction ("large/small aggregates" rather than "complete absence"). If possible, include a simple quantification of aggregate size using existing images; this should not require additional experimentation.

We updated text to clarify that we are discussing the overall state of the population and removed absolute statements including "complete absence" (Line 284). We supplemented all of our in vivo microscopy with our conjugation assays, which serves as a robust quantitative proxy measurement for aggregation.

-Line 168-171 – Please specify which mutants correspond to which processes. For example, clarify that vpsL and vpsA are linked to VPS production. This would help orient the reader.

We added identifiers of the system disrupted for each of the mutants tested (Lines 171-172).

-General comment: Different isolates of V. cholerae are used in this study. Please specify in the text which isolate was used for each experiment (e.g., was the library derived from C6706?).

We updated figure legends to specify which *V. cholerae* strains are being used in each experiment (Figures 1,2, 3, 6, S1, and S6).

-In 1A, Please clearly mark which cells are designated as assemblies versus planktonic. In the merged image, the red and green signals appear intermixed, yet the text states that they form mono communities. Please clarify.

In Figure 1A, the red and green signals are from isogenic strains expressing either mNeonGreen or mCherry. Considering this, the fact that they are intermixed is precisely the indicator that they are non-clonal monospecific (single species) aggregates rather than clusters of cells all derived from a single cell. We updated text to clarify this point (Lines 130-131).

-Strain table is nicely presented. I do recommend two additions: first, can locus tags be provided, alongside the gene names. This is helpful to community in general when following up on your work. Secondly, is the vc729 a neutral locus? Perhaps mention it somewhere in text.

We updated the strain table to include locus tags (Supplementary Table S1). VC729 is a neutral gene based on transposon mutagenesis experiments (Fu *et al*, 2013; Pritchard *et al*, 2014). We also revised text to reflect this (Line 408-409).

-Typo – KW cholerae (the e is missing).
-Line 252-253 – Please check figure call outs. They appear to be switched.

The typos identified have been corrected (Supplemental Table S1). We double checked that figure callouts throughout the manuscript are correct.

Reviewer #2 (Remarks to the Author):

The manuscript by Virgo et al. addresses the interesting topic of the dynamic spatial organization of bacterial communities and how it affects interspecies interactions. Using Vibrio cholerae and Escherichia coli in a zebrafish colonization model, they show that the presence of E. coli affects the aggregation phenotype of Vibrio by downregulating TCP pilus expression. The authors further argue that this phenotype is AI-2-dependent. As aggregation results in less T6SS-mediated killing of competing E. coli cells, disaggregation might be a mechanism to effectively attack and eliminate the competitor. This suggests that Vibrio relies on quorum sensing to balance between colonization-promoting aggregation and active killing single-cell states.

The observations are very intriguing and would be of interest to a wide readership, and the manuscript is well written. I do, however, have several points that the authors might want to consider in order to strengthen their arguments, especially regarding the AI-2 dependency of the observed phenotype:

We thank the reviewer for their positive feedback and comments to strengthen our manuscript message.

Figure 3. I think basing the conclusions solely on the luxS knockout may not be sufficient to make a strong point. The luxS deletion is notoriously pleiotropic and affects the expression of many genes in E. coli in an AI-2-independent manner (including motility and a large set of metabolic genes). This might ultimately also affect the colonization ability of the strain itself. It would therefore be useful to see the CFUs of E. coli WT and luxS in monocolonized zebrafish, as well as in co-colonization with Vibrio, to exclude colonization-rate–associated effects.

We performed monocolonization experiments with the *E. coli ΔluxS* mutant. This new data is presented in Supplementary Figure S4 (Line 229-231).

I would not exclude the luxS mutant from the analysis, but I would definitely add some control experiments. Utilizing more specific knockouts would help to modulate extracellular AI-2 levels. I suggest comparing the effects of E. coli lsrK (AI-2–overproducing) and/or E. coli lsrR (AI-2–consuming) strains.

As suggested by the reviewer, we constructed Δ*lsrR* and Δ*lsrK* mutants in *E. coli.* Preliminary experiments with these strains did not result in a noticeable difference in conjugation efficiency of *V. cholerae* compared to WT *E. coli*. On a related note, as shown in Figure 1C, when we titrate out the effect of *E. coli* on aggregation, we observe in our assay a clear thresholding effect where increasing the amount of *E. coli* above a particular amount results in complete loss of aggregation, but reducing the amount of *E. coli* below that threshold results in maximal levels of aggregation. Therefore, it is not surprising that a small change in the amount of AI-2 produced does not impact our measured amounts of aggregation. As such, to avoid further complicating the manuscript message, we elected to not include these mutants.

A luxPQ mutation in Vibrio would also be important to test in order to demonstrate that the disaggregation effect is indeed governed by AI-2.

We constructed a Δ*luxQ* mutant. In this mutant, bacteria are in a permanently disaggregated state. As such, the deletion of *luxQ* does not change *Vibrio* behavior in response to the presence of *E. coli*. These new data have been included as Supplemental Figure S3 (Lines 199-201).

Finally, I wonder if there is a possibility to show that it is indeed AI-2, and nothing else, that affects TCP expression in Vibrio. Adding exogenous AI-2 (commercially available) might be a way to test this.

We were unable to find a commercial source of AI-2 to perform this experiment. However, the reviewer's point regarding exclusivity of the aggregation modulation effect of AI-2 is well taken, especially considering our observations of the mouse isolates discussed above (see responses to reviewer 1). We therefore tempered our claims and made certain that we do not claim that AI-2 is the *only* signal modulating *V. cholerae* aggregation (Lines 265-267).

Figure 4C. I suggest swapping the panels so that E. coli WT is shown first.

We swapped the panels of Figure 4C as suggested.

Furthermore, it is somewhat difficult to see the point being conveyed ("In the absence of luxS, V. cholerae and E. coli formed distinct microcolonies of species-segregated cells (Figure 4C, Video 6). However, with WT E. coli, there were a number of dispersed individual V. cholerae cells that became intermixed among E. coli; lines 216–217"). I do agree that it looks like Vibrio forms fewer aggregates (though this somehow also seems to be the case for E. coli WT as compared to luxS), but to my eye, the populations look intermixed in both conditions. Is there a way to show it in a more pronounced way?

The message here is not that there is no intermixing of *V. cholerae* with *E. coli* in the absence of *luxS*, but rather that when *V. cholerae* is mixed with WT *E. coli*, there are now individual, non-aggregated *V. cholerae* cells that intermixed into pockets of *E. coli*. Put another way, Δ*luxS E. coli* form larger pockets of cells that do not contain *V. cholerae*. Importantly, microscopy in this case serves as motivation for performing the more quantitative CFU counting experiments presented in Figure 4D. We clarified this in the revised text (Lines 221-224, 238-240).

Figure 5. In my opinion, this argument is a bit stretched. First, it would definitely help to show the colonization levels of these species, as well as Vibrio, in your experiments. The effects might simply be due to the fact that these species successfully compete against Vibrio, preventing it from reaching the CFU/g counts that are permissive for conjugation.

Each of these isolates was collected after outgrowth on blood agar. In this case our primary goal was to demonstrate that resident microbiota of the mammalian gut could modulate *V cholerae* aggregation. We therefore performed these experiments *in vitro* to avoid complications arising from differences in colonization.

We originally omitted raw CFU counts because they did not change between different strains, but the reviewer does a make a good point that interbacterial competition could obscure conjugation results. We added these data to Figure 5 of the revised manuscript.

As all these bacteria are known to produce AI-2, you would again need to knock out luxS in at least one of them that is genetically tractable, or instead use a species that does not produce AI-2. For example, P. aeruginosa, although not normally part of the gut microbiome, can colonize the gut under certain pathological conditions.

We used an AI-2 biosensor strain to quantify AI-2 production for each of these isolates. See response to reviewer 1.

Figure 6. If I understand the authors correctly, Vibrio, by sensing E. coli, actively disaggregates to be able to kill competing cells more efficiently via T6SS. This poses a trade-off: aggregation is important for colonization, while disaggregation is important for eliminating competitors but at the same time decreases colonization. I wonder if the authors might test a Vibrio strain locked in TCP expression (e.g., from a constitutive promoter, so that its expression is not repressed in the presence of E. coli). If the authors' explanation is correct, one would expect that in this scenario, Vibrio should still be less fit compared to the wild-type strain in the presence of E. coli, as the ability to dynamically switch should yield the highest fitness.

We thank the reviewer for this suggestion. We already attempted to constitutively overexpress *tcpP*, which is known to be a positive regulator of TCP (Krukonis *et al*, 2000). However, we did not observe any changes in aggregation (our unpublished observations), probably due to there being additional post-translational controls on TCP production (Matson & DiRita, 2005).

We also constructed a ΔluxQ mutant that should be insensitive to AI-2. However, in this case we found that the base state in this mutant is in the non-aggregated form (Supplemental Figure S3).

Ultimately, given the complexity of TCP regulation in *V. cholerae*, and the multiple possible inputs into this regulatory pathway, it may not be technically possible to do the experiment suggested here. Properly exploring when and how TCP modulation occurs *in vivo* would extend beyond the aims of the present study.

Figure 1: It would be interesting to see how E. coli WT (and luxS?) localizes relative to Vibrio.

This experiment is presented in Figure 4C. The major difference between *E. coli* WT and *E. coli* Δ*luxS* when mixed with *V. cholerae* is infiltration of *V. cholerae* into pockets of growing *E. coli* (Lines 221-224).

Figure 2A: I suggest labeling the graph with “in vitro.” Same for Figures 4E and 4F. Small guiding labels would be helpful for the reader.

We updated Figures 2A, 4E, 4F in accordance with the reviewer’s suggestions.

Figure 4D. I wonder how the 2740-80 T6 vs E. coli WT group looks like.

For our original submission, we did not include this combination of strains to minimize the number of animals used. However, in our previous work (Virgo *et al.*, 2024), we performed this experiment and found that relative to 2740-80 WT vs E. coli WT, 2740-80 ΔT6 vs E. coli WT behaved the same as 2740-80 ΔT6 vs E. coli Δ*luxS*. See Figure 1D from (Virgo *et al.*, 2024).

REFERENCES

Fu Y, Waldor MK, Mekalanos JJ (2013) Tn-Seq analysis of Vibrio cholerae intestinal colonization reveals a role for T6SS-mediated antibacterial activity in the host. *Cell Host Microbe* 14: 652-663

Keizers M, Dobrindt U, Berger M (2022) A Simple Biosensor-Based Assay for Quantitative Autoinducer-2 Analysis. *ACS Synth Biol* 11: 747-759

Krukonis ES, Yu RR, Dirita VJ (2000) The Vibrio cholerae ToxR/TcpP/ToxT virulence cascade: distinct roles for two membrane-localized transcriptional activators on a single promoter. *Mol Microbiol* 38: 67-84

Matson JS, DiRita VJ (2005) Degradation of the membrane-localized virulence activator TcpP by the YaeL protease in Vibrio cholerae. *Proc Natl Acad Sci U S A* 102: 16403-16408

Pritchard JR, Chao MC, Abel S, Davis BM, Baranowski C, Zhang YJ, Rubin EJ, Waldor MK (2014) ARTIST: high-resolution genome-wide assessment of fitness using transposon-insertion sequencing. *PLoS Genet* 10: e1004782

Virgo M, Mostowy S, Ho BT (2024) Use of zebrafish to identify host responses specific to type VI secretion system mediated interbacterial antagonism. *PLoS Pathog* 20: e1012384

Reviewer #1 (Remarks to the Author):
The authors have answered my queries and done several experiments to help address questions raised by both the referees. The updated manuscript reads nice!

I only had a minor suggestion for the authors. Rather than using the phrase knocked out, consider phrasing as deletion? Or consider adding a deletion symbol in front of the strain.

We now use “delete” rather than “knock out” throughout our revised manuscript.

Reviewer #2 (Remarks to the Author):
I thank the authors for addressing all the comments. I am, however, not entirely convinced by the explanation provided as to why deletion of lsrK and lsrR did not lead to changes in conjugation rates. The accumulation and depletion of AI-2 in the supernatants of these mutants, respectively, represent quite substantial changes in overall AI-2 levels and can hardly be considered small.

We have quantified the amount of AI-2 produced by our Δ*lsrK* and Δ*lsrR* deletion mutant strains.

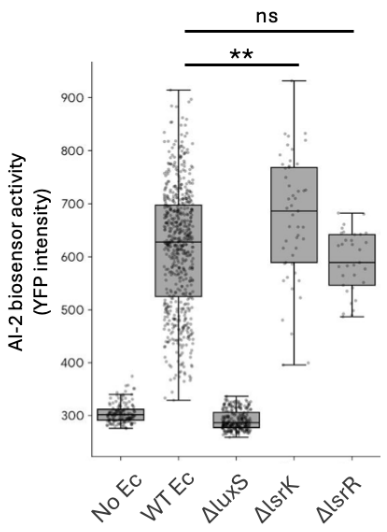

Supplementary Figure S5 (excerpt)
E. coli strains were co-incubated with an AI-2 biosensor strain. Mean fluorescence intensity of individual biosensor strain cells was then measured. AI-2 activity produced by the Δ*lsrK* mutant was slightly higher than WT ($p < 0.01$), while the difference between Δ*lsrR* and WT was not statistically significant.

Although the Δ*lsrK* mutant slightly increases the amount of AI-2 and the Δ*lsrR* mutant slightly decreases the amount of AI-2, these differences are relatively small compared to the difference between the wild type (WT) strain and the Δ*luxS* mutant. These differences may be smaller than what has been reported by others (Wang, et al. 2005. J Bacteriol.) because we are performing these assays under conditions (input cell densities, duration of experiment) similar to our conjugation/aggregation assays.

These new data have been added to Supplementary Figure S5.

Since the main message of the manuscript is that the modulation of aggregation is (mainly) regulated by AI-2, I suggest measuring extracellular AI-2 levels in the titration experiments shown in Figure 1C to provide a clearer link between AI-2 accumulation and E. coli abundance/conjugation efficiency.

The titration experiment presented in Figure 1C requires growth of both *V. cholerae* and *E. coli* over the course of 3 hours. Given that AI-2 sensing also requires growth of the biosensor strain, doing the suggested experiment may not be feasible from a technical standpoint. Moreover, considering AI-2 levels are expected to change over time, and the *V. cholerae* present will also be producing some amount of AI-2, the results will be too convoluted to properly interpret.

However, to address the spirit of the reviewer’s concern, we devised an alternative experiment to provide a clearer link between AI-2 accumulation and *V. cholerae* conjugation efficiency. We performed a titration experiment like the one presented in Figure 1C with the Δ*lsrK* and Δ*lsrR* mutants and with smaller increments. These new data have been added to Supplementary Figure S3, with corresponding discussion added to the text (lines 199-209).

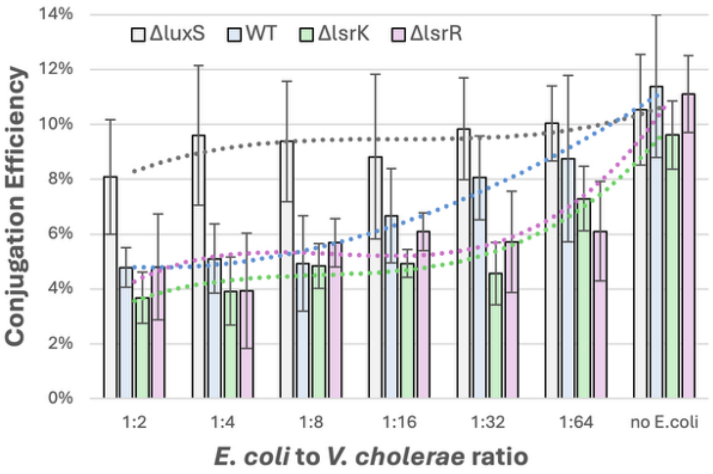

Supplementary Figure S3A
Conjugation efficiency between *V. cholerae* wild type donor (Cm^S, $J13^+$), and recipient (Cm^R) at 3 hours post-mixing. *E. coli* was titrated in at ratios of 1:2, 1:4, 1:8, 1:16, 1:32, 1:64 (*E. coli* to total *V. cholerae*). An equal volume of LB was added in the instance where no *E. coli* was added (No *E.coli*). 5-6 biological replicates were performed for each strain. Trendlines are the best fit polynomial (order 3) and are included for illustrative purposes only. Bars indicate mean ± Stdev.

These new data show that, while both Δ*lsrK* and Δ*lsrR* mutants suppress *V. cholerae* conjugation, they do so at lower dilutions compared to the wild type. Notably at the 1:32 dilution, wild type *E. coli* is not effective at suppressing conjugation, while both the Δ*lsrK* and Δ*lsrR* mutants are. Why both mutants have a similar effect is not yet clear – perhaps *V. cholerae* is able to sense both the excess and depletion of AI-2 – and fully resolving how *V. cholerae* senses interspecies signals like AI-2 will be the subject of future work. Considering a direct link between AI-2 and *V. cholerae* biofilm formation has already been established (for example, Bridges and Bassler, 2019, PLoS Biology), working out this mechanism is not central to the main message of our manuscript, namely that *V. cholerae* can sense potential prey bacteria and change its community structure accordingly to better compete with them.

I would also recommend including the data from the lsrK/lsrR mutants as supplementary material, and incorporating these results into the discussion.

These new data have been added to Supplemental Figure S3, with corresponding discussion added to the text (lines 199-204).
